# Increased migration and motility in XIAP-null cells mediated by the C-RAF protein kinase

**DOI:** 10.1038/s41598-022-11438-8

**Published:** 2022-05-13

**Authors:** Lauren G. Russell, Lydia A. K. Davis, Jill E. Hunter, Neil D. Perkins, Niall S. Kenneth

**Affiliations:** 1 Newcastle University Biosciences Institute, Faculty of Medical Sciences, Newcastle University, Newcastle upon Tyne, NE2 4HH UK; 2 Department of Molecular Physiology and Cell Signalling, Institute of Systems, Molecular and Integrative Biology, University of Liverpool, Liverpool, L69 7ZB UK

**Keywords:** Oncogenes, Apoptosis, Collective cell migration

## Abstract

The product encoded by the X-linked inhibitor of apoptosis (XIAP) gene is a multi-functional protein which not only controls caspase-dependent cell death, but also participates in inflammatory signalling, copper homeostasis, response to hypoxia and control of cell migration. Deregulation of XIAP, either by elevated expression or inherited genetic deletion, is associated with several human disease states. Reconciling XIAP-dependent signalling pathways with its role in disease progression is essential to understand how XIAP promotes the progression of human pathologies. In this study we have created a panel of genetically modified XIAP-null cell lines using TALENs and CRISPR/Cas9 to investigate the functional outcome of XIAP deletion. Surprisingly, in our genetically modified cells XIAP deletion had no effect on programmed cell death, but instead the primary phenotype we observed was a profound increase in cell migration rates. Furthermore, we found that XIAP-dependent suppression of cell migration was dependent on XIAPdependent control of C-RAF levels, a protein kinase which controls cell signalling pathways that regulate the cytoskeleton. These results suggest that XIAP is not necessary for control of the apoptotic signalling cascade, however it does have a critical role in controlling cell migration and motility that cannot be compensated for in XIAP-knockout cells.

## Introduction

**Figure 1:**
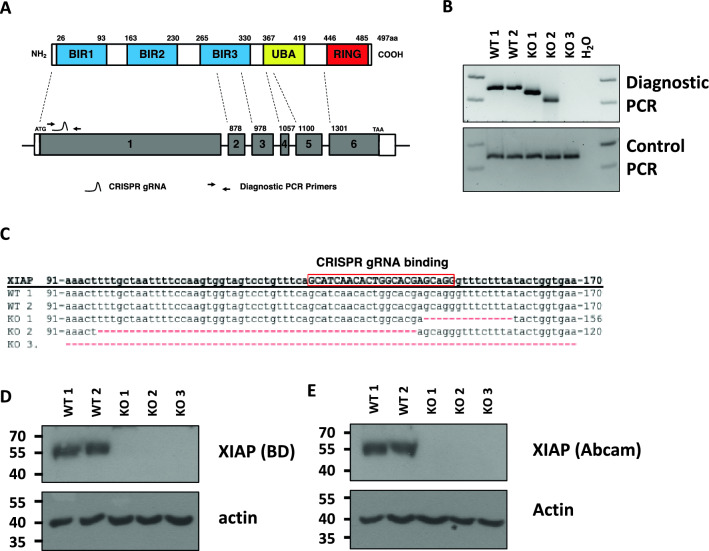
Creation of XIAP null cell lines. (**A**) Schematic of the exon structure of the XIAP gene and the domains of the XIAP protein. Location of the CRISPR gRNA binding site and the diagnostic PCR primers are indicated. (**B**) PCR analysis of genomic DNA prepared from U2OS clonal cell lines prepared from CRISPR/Cas9 transfected cells. (**C**) Alignment of the sequencing data of XIAP exon 1 from clonal cells lines. Whole cell lysates were prepared from CRISPR/Cas9 monoclonal U2OS cells. Lysates were subjected to immunoblot analysis to assess XIAP expression levels using (**D**) an XIAP antibody from BD (immunogen amino acids 268–426) and (**E**) an Abcam XIAP antibody (immunogen amino acids 352–449).

The Inhibitors of apoptosis (IAP) proteins are a family of functionally and structurally related proteins predominantly known for the regulation of caspases and immune signalling^1^. In mammalian cells there are eight IAP family members all characterised by the presence of at least one baculovirus repeat (BIR), the domain utilized by viral IAP proteins to compromise host cell death machinery^2^. Of the 8 mammalian IAP family members the X-linked IAP (XIAP) is perhaps the best-characterized, with the most direct links to human disease^3,4^. XIAP contains 3 BIR domains located on the amino terminus of the protein that allow XIAP to inhibit apoptotic cell death. In fact XIAP is the only mammalian IAP protein that has the capacity to directly bind to and functionally inhibit caspases; specifically caspase-3, caspase-7 and caspase-9, to suppress apoptosis^5–7^. In addition to the BIR domains XIAP contains a carboxy-terminal RING domain that provides it with E3 ubiquitin ligase activity, as well as a ubiquitin associated domain (UBA) that can interact with ubiquitin chains (Fig. 1A)^8–11^. This enables XIAP to participate in multiple ubiquitin-dependent signal transduction cascades enabling it to act as a key intermediate in a variety of cellular pathways including, but not limited to: NF-κB signalling^3,12,13^, MAPK/JNK signalling^14^, maintenance of intracellular copper levels^15–17^, hypoxia-induced gene expression^18^, Wnt/bcatenin signalling^19^, regulation of autophagy^20–22^ and control of cell motility and migration^23–28^. Due to its key role in signal transduction pathways, deregulation of XIAP has been implicated in the pathogenesis of human cancers and inflammatory diseases^3^. XIAP is highly overexpressed in many forms of cancer, including breast^29^, renal^30,31^, bladder^32^ and certain haematological malignancies^33^. Indeed, a systematic review correlates high XIAP expression and poor patient outcomes in a variety of solid tumours^34^. Conversely, inactivating genetic mutations in the XIAP gene can result in an extremely rare primary immunodeficiency in humans, Type 2 X-linked lymphoproliferative disease (XLP2), characterised by a defective immune system that is powerfully responsive to infection with the Epstein-Barr virus (EBV)^4,35,36^. It is therefore important to reconcile the molecular properties of XIAP with its role in the progression of human disease states to understand the critical roles of XIAP, so as to facilitate the development of novel therapeutics.

To study the cellular function of XIAP we created XIAP-null human cell lines using site directed nucleases and compared their properties with isogenic wildtype controls. Surprisingly, we find no obvious defects in the induction of caspase-dependent apoptosis in XIAP-deficient cell lines. Instead, the primary XIAP-dependent defect we observe is an increase in cell migration in XIAP-null cells. We find that levels of C-RAF protein kinase are elevated in XIAP-null cells, consistent with increased cell migration rates. Re-expression of XIAP was sufficient to reduce C-RAF levels and suppress the motility of XIAP-null cells. However, introduction of an XIAP mutant that does not have E3 ligase activity did not reverse the invasive phenotype, indicating that control of cell migration by XIAP relies on its ability to ubiquitinate substrates. Together our results suggest that XIAP is critical for modulating cell motility in a manner dependent on its ability to conjugate ubiquitin to target substrates.

## Materials and Methods

### Cells

U2OS and HeLa cells were sourced from the ATCC and grown in DMEM supplemented with 10% FBS, 1% L-gluatamine, and 1% penicillin streptomycin at 37 °C and 5% CO2. Cells were routinely tested for contamination.

### Plasmids

pEBB-HA, pEBB-HA-XIAP, pEBB-HA-XIAP-D148A/W310, pEBB-HA-XIAP-F495A were kind gifts from Colin Duckett (Duke University). CRISPR/Cas9 and TALEN constructs are described in the relevant sections.

### CRISPR/Cas9 genome editing

CRISPR/Cas9 gRNAs were designed using using zifit.partners.org. The oligos 5’ ACACCGCATCAACACTGGCACGAGCG 3ʹ and 5ʹ AAAACGCTCGTGCCAGTGTTGATGCG 3ʹ were annealed and cloned into the vector MLM3636 (Addgene #43,860) cut with BsmBI. The CRISPR gRNA plasmid was transfected with the JDS246 (Addgene plasmid #43,861) a mammalian codon optimized Cas9 nuclease with C-term 3X FLAG, using Genejuice transfection reagent (Merck) according to the manufacturer’s instructions. Clonal cell lines were isolated by limiting dilution. Genomic DNA was isolated using DirectPCR lysis buffer (Viagen). Gene disruption was analysed by PCR using primers spanning the CRISPR gRNA binding region Sense 5ʹ- aaagtctgttgcttgtgtttca- 3ʹ and Antisense 5ʹ taccagaatttgtagactgcgt- 3ʹ and a PCR control region downstream of the CRISPR gRNA binding site to check quality of the genomic DNA preparations. Sequencing reactions were performed using the custom primer 5ʹ tgacaactaaagcaccgcac 3ʹ.

### TALEN genome editing

TALENS pairs were designed using the algorithm on zifit.partners.org. TALE repeats were created by nested PCR and cloned into the FokI containing plasmids JDS71 (Addgene #32,285) for the Left TALEN (binding to the DNA sequence TCCTGTTTCAGCATCAAC) and JDS70 (Addgene #32,285) for the Right TALEN (binding to the DNA sequence TTCACCAGTATAAAGAAA). The TALEN pair is predicted to create a DNA double strand after the base pair 145 within exon 1 of the XIAP gene, corresponding to amino acid Arginine 48. TALEN transfections were performed using the Amaxa nucleofector, using the nucleofector kit V using the X-001 program. Clonal cell lines were isolated by limiting dilution and characterized using the same primer pairs used for the CRISPR/Cas9 lines.

### Wound healing assay

Cells were seeded onto 6 well plates at a density of 25,000 cells/well and incubated at 37 ºC for 72 h. A scratch was made across the well using a sterile pipette tip and cultured in fresh media. A line was drawn perpendicular to the scratch and an image was taken of the scratch underneath the line using an EVOS XL Core microscope. Plates were marked perpendicular to the scratch to ensure the same area was imaged at each time point. The wounds were imaged at 0, 6, 24 and 28 h after the scratch was made. The area of the wound was measured at each time point using ImageJ software and normalised to 100% at time 0. The data were plotted using GraphPad PRISM and the area underneath the curve was quantified. Each data point represents the area calculated from the closure of a single scratch. Statistical analysis was performed by Student’s T-test or one-way ANOVA with Dunnett’s multiple comparisons test. Where specified, the area under the curve was calculated and analysed using this method. A value of *P* < 0.05 was considered to be statistically significant.

### Cell viability studies

Cells were seeded into 96 well plates at a density of 5000 cells/well and incubated at 37 ºC overnight. Etoposide (Sigma) or doxorubicin (Sigma) was diluted in supplemented DMEM to achieve a gradient of drug concentrations as specified in the figure legend. This was incubated at 37 ºC for 48 h, after which the media was removed and 100 μl of 5% PrestoBlue® (Invitrogen) diluted in supplemented DMEM was pipetted into each well. After 2 h’ incubation at 37 ºC, 90 μl from each well was pipetted into a new 96 well plate and the fluorescence intensity was recorded at 570 nm using POLAR star Omega plate reader. The cell viability was expressed as a percentage relative to untreated controls.

### Immunoblotting and antibodies

Immunoblot analysis was performed as described in^37^. Briefly, cell lysates were prepared with modified RIPA lysis buffer (50 mM Tris pH 8.0, 150 mM NaCl, 1% NP40, 0.5% C_24_H_39_NaO_4_, 0.1% SDS) supplemented with protease inhibitors and incubated on ice for 15 min. Lysates were then sonicated in a Biodisrupter UltraSonic Waterbath (Diagenode) (2 × 30 s cycles on high) to shear the genomic DNA and clarified by centrifugation at 13 000 rpm at 4 °C for 10 min. Protein concentration was determined by BCA assay and 20 μg of protein was loaded per well. Immunoblotting was performed using the following antibodies: β-actin (AC74, Sigma), XIAP (610,762, BD Transduction Laboratories), XIAP (ab28151, Abcam), cIAP1 (1E1-1–10, Enzo Life Sciences), C-RAF (A301-519A, Bethyl Laboratories) Rac1/Cdc42 (#4651, Cell Signalling Technologies) and Actin (Ac74, Sigma).

### Caspase activity assay

Cells were seeded into 96-well plates at a density of 5000 cells/well. The following day cells were treated as indicated and the caspase activity was determined using Caspase-Glo assay (Promega) according to the manufacturer's protocol. Raw RLU values are presented and statistical analysis performed using a one-way ANOVA with Dunnett’s multiple comparisons test. A value of *P* < 0.05 was considered to be statistically significant.

### Proliferation experiment

Clonal cell lines were seeded into 96 well plates at a density of 2500 cells/well. The plates were incubated at 37 ºC and measured using 5% PrestoBlue® (as described above) after either 24, 48, 72 or 96 h incubation. The gain was determined after 24 h and was subsequently used to measure all plates to allow for comparison between time points. Raw values were plotted to indicate cell growth/proliferation over time.

### siRNA transfection

siRNA duplex oligonucleotides were synthesised by MWG and transfected using Interferin (Polyplus) according to the manufacturer's instructions. siRNA sequences: control—CAG UCG CGU UUG CGA CUG G; XIAP —GUG GUA GUC CUG UUU CAG C.

### Statistical analysis

Statistical analysis was performed by a one-way ANOVA with Dunnett’s multiple comparisons test or unpaired Student’s t-test where appropriate. Where specified, the area under the curve was calculated and analysed using these methods. If a *P*-value is less than 0.05, it is flagged with one star (*). If a *P*-value is less than 0.01, it is flagged with two stars (**). If a *P*-value is less than 0.001, it is flagged with three stars (***).

## Results

### Creation of XIAP-null cell lines

To disrupt the coding region of the XIAP gene a CRISPR guide RNA (gRNA) was designed to bind within the first exon of the XIAP gene (Fig. 1A). Plasmids encoding the CRISPR gRNA and the Cas9 nuclease were transfected into U2OS cells to induce DNA double strand breaks (DSBs) and disrupt the XIAP coding region (Fig. 1A). DBSs were induced 150 bp downstream from the start site of translation, corresponding to glycine 50, within the first BIR of the XIAP protein (Fig. 1A). PCR analysis was performed on genomic DNA isolated from clonal cell lines using specific primers flanking the site of DSBs to assess targeted gene disruption (Fig. 1A,B). Only clones with a single PCR product, indicating homozygosity, were taken forward for further analysis. Three disrupted clones were identified; two with PCR products smaller than the wildtype (small deletion of the XIAP gene) (KO 1 and KO 2) and one where the PCR product was absent (large deletion of the XIAP gene) (KO 3) (Fig. 1B). The absence of PCR product from KO 3 was not due to lack of genomic DNA in the reaction, as control PCR primers amplifying an unrelated region of the XIAP coding sequence was efficiently amplified (Fig. 1B). Rather it was predicted that a primer binding site was deleted from exon 1. DNA sequencing analysis revealed a 14 bp deletion in clone KO 1 and a 50 bp deletion in clone KO 2, which both result in frameshift mutations within the first exon of the XIAP gene (Fig. 1C). Immunoblot analysis, using 2 different anti XIAP monoclonal antibodies, confirmed XIAP deletion in clones KO 1 and KO 2 and revealed that the large deletion of the XIAP gene in clone KO 3 also resulted in the creation of a XIAP-null cell line (Fig. 1D,E). Together these results confirm 3 distinct genetic disruptions of the XIAP gene, allowing us to assess the effects of XIAP deletion in cells.

### XIAP null cells do not have increased sensitivity to cell death

**Figure 2:**
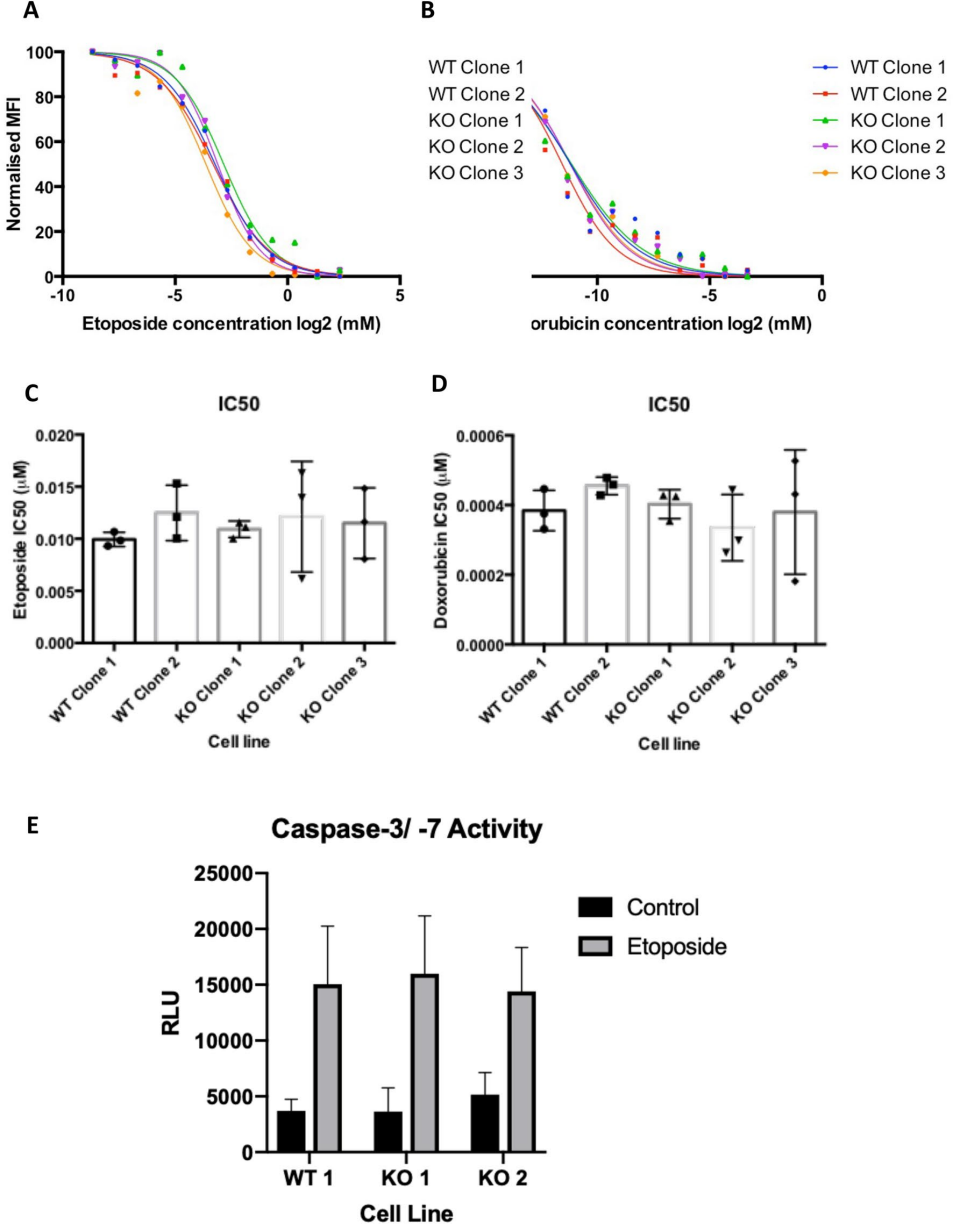
XIAP status does not alter cell viability in response to cytotoxic agents. (**A**) U2OS clonal cell lines were exposed to etoposide for 48 h and viability was determined by PrestoBlue assay. The normalised mean fluorescence intensity (MFI) at increasing concentrations of etoposide (log2(mM)) of three independent experiments for each clone is averaged and plotted (Individual repeats shown in Supp Fig. 1A). Statistical analysis performed using a one-way ANOVA with Dunnett’s multiple comparisons test. (**B**) As in A but using doxorubicin. (**C**) The mean normalised IC50 (mM) for etoposide is plotted for each clone. (**D**) As in C. but for doxorubicin. (**E**) U2OS clonal cell lines treated with 50uM Etoposide for 24 h as indicated. Caspase-3 -7 activity was determined by Caspase-Glo assay and values normalised to untreated controls. Error bars represent standard deviation. Statistical analysis performed using a one-way ANOVA with Dunnett’s multiple comparisons test.

As XIAP is best described as a suppressor of caspase-dependent cell death we initially examined if XIAP-null U2OS cells are more sensitive to genotoxic stress induced by DNA damaging agents. Two wildtype and three independent isogenic XIAP-null cell lines were treated with increasing doses of the DNA topoisomerase II inhibitor, etoposide, or the DNA intercalating agent, doxorubicin, both potent inducers of apoptotic cell death. Depletion of XIAP levels by RNAi has previously been reported to increase caspase-dependent apoptotic cell death in response to these DNA damage inducing agents in cancer cell lines^38^. Both etoposide and doxorubicin both efficiently induced cell death in U2OS cells (Fig. 2A,B, Supplemental Fig. 1). Surprisingly, no differences in sensitivity were observed that correlated with XIAP status of the cell lines (Fig. 2A,B, Supplementary Fig. 1). Indeed, the IC50 values calculated for both etoposide and doxorubicin showed no significant differences between any of the XIAP-wildtype or XIAP-null cell lines tested (Fig. 2C,D).

Our results indicate no differences in cell viability associated with XIAP status. However, cell viability assays do not specifically measure caspase activity associated with apoptotic cell death, but can in fact be influenced by the cumulative effects of alternative cell death pathways, cell growth rates and cell metabolic activity. As XIAP’s role in cell death control is through its ability to bind to, and inhibit, the activity of cell death effector caspases we directly measured the activities of caspases -3 and -7 in XIAP-null cell lines in response to etoposide. XIAP wildtype and XIAP-null U2OS cells were treated with etoposide and apoptotic cell death was determined using a luminescent assay directly measuring caspase3 and caspase-7 activity (Fig. 2E). Consistent with cell viability results, no significant differences in caspase activity in response to etoposide were observed between XIAP wildtype and null U2OS cell lines, indicating that loss of XIAP is not sufficient to alter either cell death or caspase activity in U2OS cells (Fig. 2E). Together our data suggests that loss of XIAP has no effect on apoptotic cell death in response to DNA damaging agents in our genetically modified cell lines.

### XIAP-null cells have increased migratory capacity

XIAP has the ability to act as an E3 ubiquitin ligase and as such is an important regulator of several cell signaling pathways^10,28,39^. Several studies have identified IAP proteins, including XIAP, as regulators of the cytoskeleton^28^. However, there have been conflicting reports on the specific role of XIAP in regulating cell motility. There is data suggesting that elevated XIAP promotes cell motility^23,26^ and other reports suggesting that high XIAP suppresses migration rates in cultured cell lines^24,25^. We therefore decided to use XIAP-null cells to investigate if genetic deletion of the XIAP gene alters migration rates in U2OS cells.

**Figure 3:**
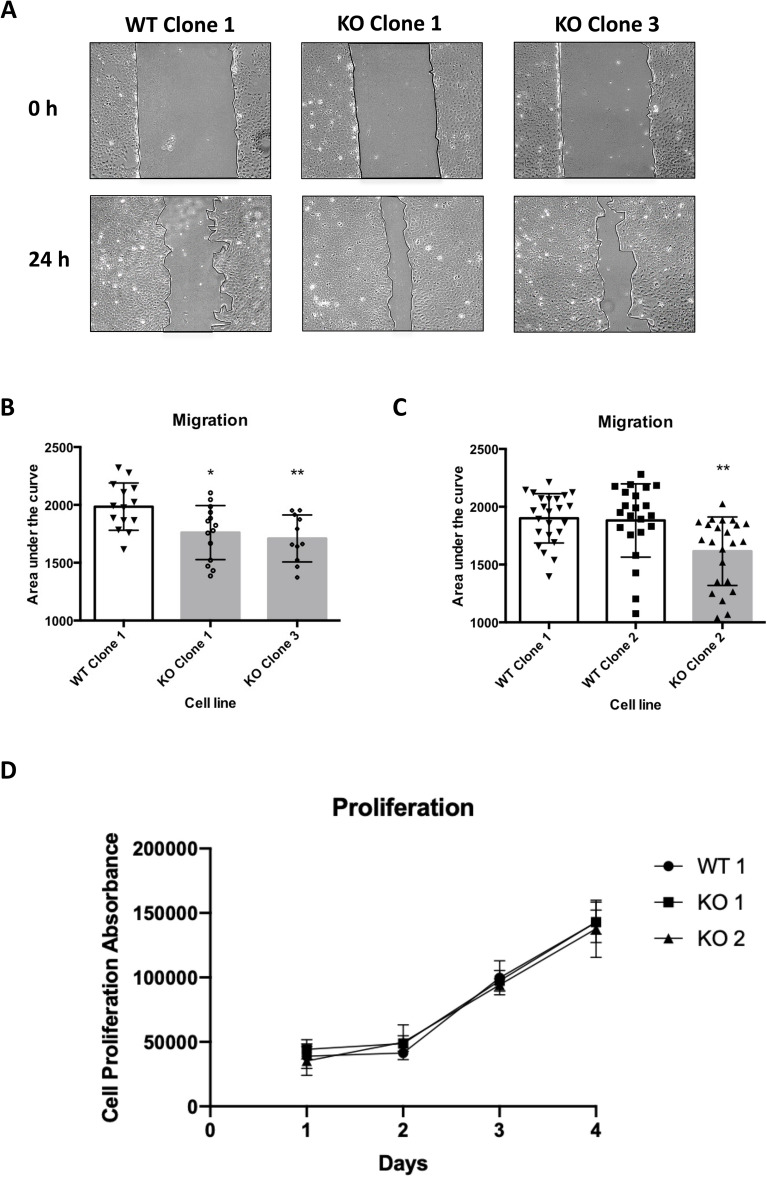
XIAP-null cells have increased migratory capacity. (**A**) Representative images of a wound healing experiment for wild type and XIAP null clones at 0 and 24 h. The migration front is highlighted for clarity. (**B**) The area of the wound was imaged and measured at 0, 8, and 24 h using ImageJ software. The data was normalised with the percentage wound area equalling 100% at 0 h. The % wound area was plotted against time and the area under the curve (% wound area × time(hours)) was calculated and analysed by one-way ANOVA using Dunnett multiple comparisons test in comparison to the wildtype clone 1. Each measurement represents data from an individual scratch. Statistical analysis performed using a one-way ANOVA with Dunnett’s multiple comparisons test (**C**) As in B. using independent XIAP-null clonal cell lines. (**D**) Proliferation assay performed using Prestoblue assay. Cell lines and measurement times as indicated.

XIAP wildtype and null U2OS cell lines were seeded and allowed to grow to confluency before a scratch was created using a sterile pipette tip. Multiple images of the scratch were taken over the course of the assay and area was mapped and measured using ImageJ software. The data indicate that genetic deletion of XIAP increased the migration rates, as indicated by the enhanced closure rates of the wounds in XIAP-null cells (Fig. 3A and Supplementary Fig. 2A). To quantify the migratory capacity of each clonal cell line area under the curve was calculated to map wound closure rates and XIAP deletion resulted in a significantly increased cell migration rate in each of the cell lines tested (Fig. 3B,C). To ensure that the increased closure of the wound healing assays in XIAP-null cells was indeed due to increased cell motility, and not increased cell growth/doubling time, cell proliferation rates were compared between wild type and XIAP-null cells (Fig. 3D). No differences were seen in growth rates between wildtype and XIAP-null cells indicating that differences in wound closure were indeed due to differences in cell motility rather than an increase in cell number (Fig. 3D). Indeed, quantitative and qualitative analysis showed increased morphological changes and motility, in XIAP-null cells by measuring the dynamic nature of the migration front (Supplementary Fig. 2B,C).

Like all genome editing technologies CRISPR/Cas9 can induce double strand breaks at off-target sites within the genomes of targeted cells^40^. To ensure the XIAP-dependent migration phenotype was in fact due to the targeted disruption of the XIAP gene, rather than an off-target effect of the CRISPR gRNA, we tested an independent XIAP-null cell line created using transcription activator-like effector nucleases (TALENs), an alternative genome editing tool. Plasmids encoding a TALEN pair were designed to induce a double strand break within the first exon of XIAP, 145 bp downstream of the start site of translation corresponding to arginine 48 of the XIAP protein (Supplementary Fig. 3A). Clonal cell lines were isolated by limiting dilutions and analysed by PCR (Supp. Fig. 3B). A clone homozygous for a deletion in the XIAP coding sequence was identified (Supplementary Fig. 3B). Sequencing analysis confirmed a 10 bp deletion of the XIAP coding sequence, predicted to result in a frame shift and premature stop codon (Supplementary Fig. 3C). XIAP deletion was confirmed by immunoblot analysis (Supp. Fig. 3D). Cell motility was measured using the TALEN-XIAP null cell line and compared to a matched wildtype control (Supplementary Fig. 3E). TALEN-mediated XIAP deletion resulted in extended morphology and increased cell motility, consistent with the results observed in the CRISPR/Cas9 XIAP-null cell lines (Supplementary Fig. 3D,E). To investigate if our results were cell line specific we next tested the migratory capacity of HeLa cells in which XIAP is depleted by RNAi-mediated knockdown to investigate if our effects were observed across cell lines (Supplementary Fig. 4A). Consistent with results from genetically engineered cells XIAP depletion resulted in increased migratory capacity as measured by wound healing assay (Supplementary Fig. 4B,C). Together our results indicate that XIAP deletion increases the migratory capacity of culture cells independently of the method used to create them.

### cIAP depletion does not significantly alter cell migration rates in U2OS cells

**Figure 4:**
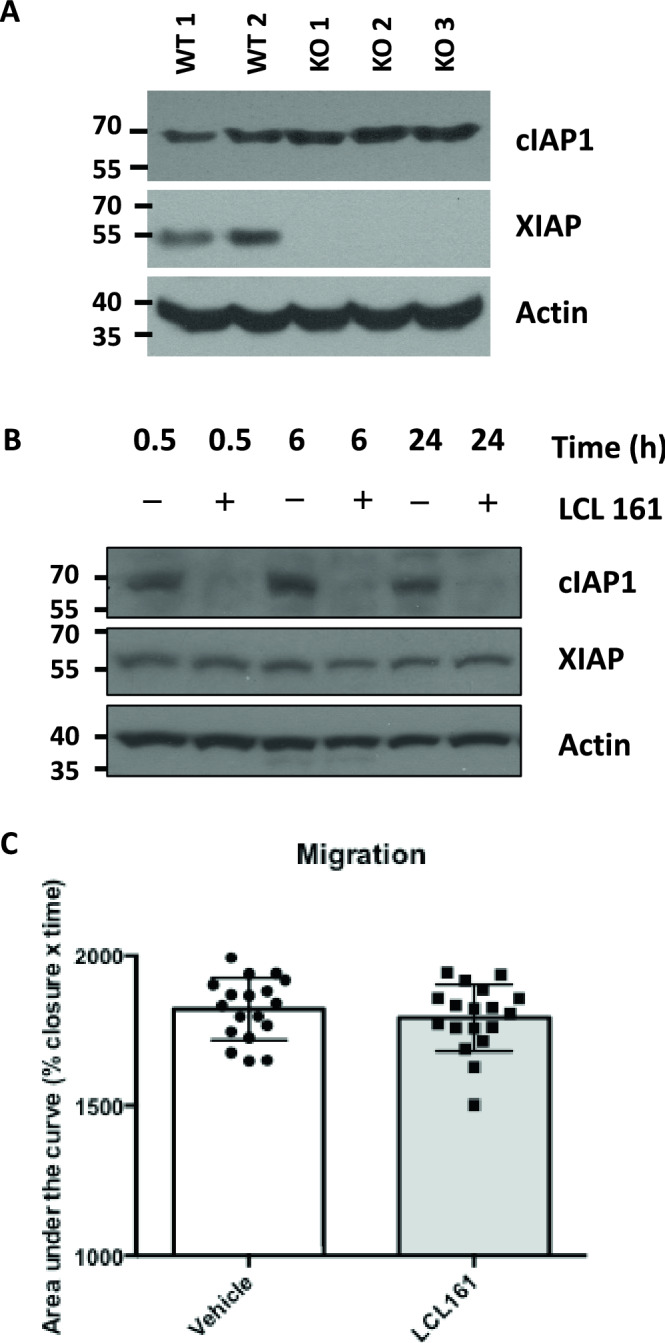
cIAP proteins does not significantly alter U2OS cell motility. (**A**) Whole cell lysates were prepared from CRISPR/Cas9 monoclonal U2OS cells. Lysates were subjected to immunoblot analysis to assess cIAP1 levels. (**B**) Wildtype U2OS cells were treated with the SMAC mimetic compound, LCL161, for the indicated times. Whole cell lysates were prepared and analysed by immunoblot analysis using the indicated antibodies. (**C**) Wound healing assays were performed on untreated and LCL161 treated U2OS cells and measured at 0, 8 and 24 h. The area of the wound was imaged and measured at 0, 8, and 24 h using ImageJ software. The data was normalised with the percentage wound area equalling 100% at 0 h (6 technical repeats of 3 independent experiments). The % wound area was plotted against time and the area under the curve (% wound area × time(hours)) was calculated and analysed by unpaired students t-test.

The related cellular IAP (cIAP) proteins, cIAP1 and cIAP2, have also been implicated in altering cell migration rates in cultured cell lines^23,26^. As deletion of IAP genes can influence the protein levels of other family members through feedback mechanisms we analysed cIAP levels in XIAP-null cell lines^41^. A modest, but insignificant, increase in cIAP1 is observed in XIAP null cells consistent with the feedback loop observed in other cellular systems (Fig. 4A, Supplementary Fig. 5A). To examine the potential roles of cIAP proteins in cell migration, independently from XIAP, U2OS wildtype cells were treated with the SMAC mimetic compound, LCL 161^42^. SMAC mimetics, such as LCL 161, although designed to bind to XIAP have been shown to target both cIAP1 and cIAP2 for proteasomal mediated degradation, without altering XIAP levels^43,44^. Indeed, immunoblot analysis of U2OS cell lysates prepared from cells treated with LCL161 revealed a rapid and specific reduction of cIAP1, which was observed as quickly as 30 min after treatment and persisted for the course of the migration experiment (Fig. 4B). As we could suppress cIAP1 levels, without altering XIAP, we measured cell migration rates in LCL161 treated cells. Cell migration was not significantly altered in cells treated with LCL161 (Fig. 4C). The data suggest that chemical depletion of cIAP proteins does not alter cell migration rates in U2OS cells.

### XIAP E3 ubiquitin ligase activity is required to suppress cell migration

**Figure 5:**
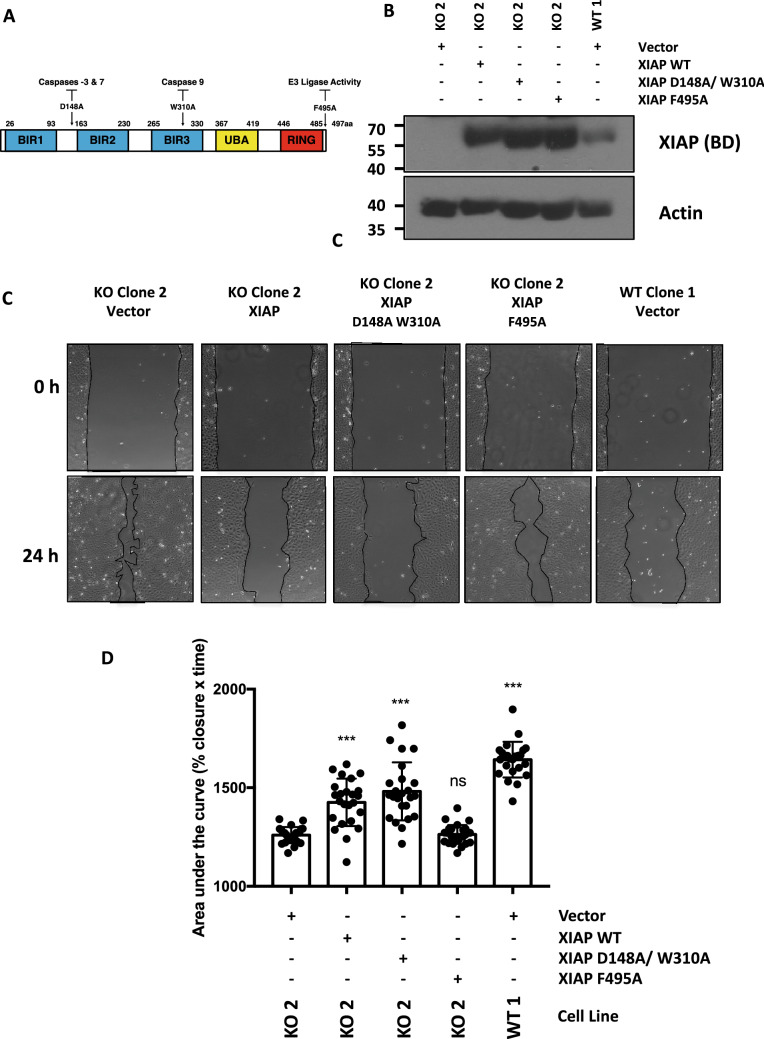
The ubiquitin ligase activity of XIAP is necessary to control cell migration rates. (**A**) Schematic of XIAP protein indicating key residues essential for mediating the caspase interactions and the E3 ubiquitin ligase activity. (**B**) Representative images of a wound healing experiment using XIAP KO clone 2 transfected with empty vector, wildtype XIAP, caspase binding mutant XIAP (D148A/W310A), and ubiquitin ligase mutant XIAP (F495A), and XIAP WT clone 1 transfected with empty vector at 0 and 24 h. The migration front is highlighted for clarity. (**C**) Whole cell lysates prepared from transfected cells and immunoblotted with XIAP and actin antibodies to determine transfection efficiency. (**D**) The area of the wound was imaged and measured at 0, 8, and 24 h using ImageJ software. The data was normalised with the percentage wound area equalling 100% at 0 h (6 technical repeats of 3 independent experiments). The % wound area was plotted against time and the area under the curve (% wound area × time(hours)) was calculated and analysed by one-way ANOVA using Dunnett multiple comparisons test in comparison to the XIAP KO clone 2 transfected with empty vector.

The XIAP protein has dual enzymatic activities, by virtue of its ability to bind caspases via its BIR domains and its E3 ubiquitin ligase activity conferred by the C-terminal RING domain (Fig. 5A). To investigate what properties of XIAP are necessary for the control of cell migration in U2OS cells, wildtype XIAP and XIAP variants were transfected into XIAP null cell lines. Based on the crystal structures of XIAP, specific point mutations have been characterized that can alter specific properties of the protein^5,6^. To examine the properties of XIAP required for its ability to regulate cell migration we reconstituted XIAP null cells with wildtype XIAP, a caspase 3/7/9 binding mutant (D148A/W310A)^45,46^ or an E3 ubiquitin ligase mutant (F495A)^47^ (Fig. 5A). Immunoblot analysis revealed that each XIAP variant was expressed to equivalent levels in XIAP-null cells (Fig. 5B). Cells reconstituted with wildtype XIAP or the caspase-binding mutant (D148A/W310A), significantly inhibited the migration of XIAP deficient cells as compared to the XIAP-deleted control cell line transfected with empty vector (Fig. 5C,D). However, reconstitution with the XIAP ubiquitin ligase mutant, F495A, failed to reverse the increased migration phenotype, indicating that the ubiquitin ligase activity of XIAP is essential for its ability to regulate cell motility in cells (Fig. 5C,D).

### XIAP regulates levels of the C-RAF protein kinase to regulate cell motility

**Figure 6:**
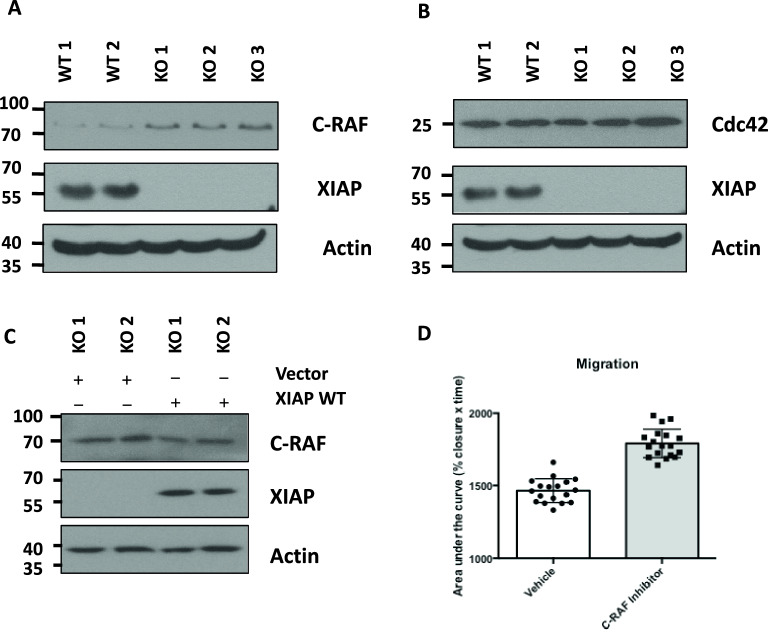
XIAP controls levels of C-RAF to influence cell migration. (**A**, **B**) Whole cell lysates were prepared from CRISPR/Cas9 monoclonal U2OS cells and immunoblotted using the indicated antibodies. (**C**) XIAP KO U2OS cells were transfected with XIAP or empty vector. Whole cell lysates were prepared and immunoblotted using the indicated antibodies. (**D**) The area of the wound of XIAP null cells (KO 2) treated with the C-RAF inhibitor, BAY 43–9006, was imaged and measured at 0, 8, and 24 h using ImageJ software. The data was normalised with the percentage wound area equalling 100% at 0 h. Each measurement represents data from an individual scratch (6 technical repeats of 3 independent experiments). The % wound area was plotted against time and the area under the curve (% wound area × time(hours)) was calculated and analysed by unpaired students t-test.

Previous studies have shown that XIAP can suppress cell migration by binding to and ubiquitinating C-RAF and Cdc42, key proteins involved in cell motility, marking then for proteasomal degradation^26,48^. C-RAF, a serine/threonine kinase in the MAP kinase signaling cascade, is a well-described promotor of cell migration and motility, and Cdc42 is a RhoGTPase that controls filopodia formation and cell migration^23,26,48^. As our data suggested that XIAP suppresses migration rates we examined levels of C-RAF and Cdc42 in the XIAP null cells. Immunoblot analysis of cell lysates prepared from XIAP wildtype and XIAP null cell lines indicate that XIAP null cells have elevated levels of C-RAF protein, but not elevated levels of Cdc42 (Fig. 6A,B, Supplementary Fig. 5B,C). To investigate if expression of XIAP could reduce C-RAF levels, XIAP null cells were transfected with plasmids encoding wildtype XIAP or empty vector. Expression of XIAP reduced levels of C-RAF compared those observed in XIAP null cells (Fig. 6C, Supplementary 5C). Our data indicate that C-RAF levels, but not Cdc42, are elevated in XIAP-null cells, and C-RAF levels can be reduced by re-introducing wildtype XIAP (Fig. 6C).

Therefore, we decided to investigate the role of C-RAF activity in regulating the rates of migration and motility. To suppress C-RAF activity XIAP-null cells were treated with the C-RAF kinase inhibitor, BAY 43–9006^49^. XIAP wildtype and knockout cells were pretreated BAY 43–9006, and cell migration rates were determined by wound healing assay compared to cells treated with vehicle alone. The migration rates of XIAP-null cells was significantly suppressed by the C-RAF inhibitor, indicating that C-RAF levels and activity were at least in part responsible for the increased migration rates in the XIAP-null cells (Fig. 6D).

Taken together these results indicate that XIAP deletion results in enhanced cellular migration rates. We find that it is the ubiquitin ligase activity of XIAP is important for the migration phenotype, through regulation of the levels of the CRAF protein kinase. In addition, we show that chemical inhibition of C-RAF reverses the enhanced cell migration observed in XIAP-null cells.

## Discussion

The results of the present study indicate that XIAP plays an important role in controlling cell migration rates. Our data, using CRISPR/Cas9 and TALEN modified cell lines demonstrate that XIAP deletion increases the motility of U2OS cells, by controlling the levels of the C-RAF protein kinase. Reconstitution of our XIAP-null cell lines with wildtype XIAP, and XIAP variants, suppress C-RAF levels and reduce cell motility, in a manner dependent on XIAP ubiquitin ligase activity. C-RAF protein levels are elevated in XIAP-null cells and reduced when XIAP is reexpressed. Importantly, treatment of XIAP-null cells with a C-RAF kinase inhibitor is sufficient to supress the increased cell migration rates.

XIAP is a well-described inhibitor of caspase-3, -7 and -9 activity, with overexpression of the XIAP protein a well-defined suppressor of cell death^5,7,29,39^. As such a number of drugs that directly target XIAP are in development and these inhibitors might enhance chemosensitivity in XIAP over-expressing tumours^50–52^. However, the cellular role of endogenous XIAP in the regulation apoptotic cell death is less clear. The XIAP-null mouse is born at expected Mendalian ratios with no obvious cell death related phenotype^53^. Instead, many studies have indicated that a key role for the IAP family of proteins is to act as E3 ubiquitin ligases to regulate multiple cellular processes. Indeed, XIAP deficiency can result in more aggressive disease in a murine prostate cancer model which suggests a degree of caution to be employed before XIAP antagonists are used for cancer therapy^54^.

Several studies have previously demonstrated a link between XIAP and cIAP levels and cell motility/migration^28^. XIAP deletion or depletion has been reported to increase cell migration and motility through increasing levels of C-RAF and Cdc42^23,26^. However, conflicting reports have demonstrated that XIAP can negatively regulate RhoGDI activity by modulating its SUMOylation, thereby increasing actin polymerization and cell motility, resulting in XIAP depletion causing decreases in cell migration^24,55,56^. These conflicting results may arise by localised differences of the downstream IAP signalling molecules in each individual cell type. Results from cell lines used our study show that XIAP deletion increases levels of C-RAF, and C-RAF inhibition reduced the migratory capacity of XIAP deficient cells lines.

Our data show that XIAP dependent control of cell migration is at least in part due to its ability to modulate the levels of the C-RAF protein kinase. A major challenge in understanding XIAP-deficient disease is reconciling the cellular properties of XIAP with the symptoms associated disease. One of the major symptoms associated with XIAP deficiency is the development of severe haemorrhagic colitis, which is associated with its key role in inflammatory signalling through the NOD signalling pathway^57–60^. However, XIAP-deficient patients also present with hemophagocytic lymphohistiocytosis (HLH), recurrent fevers, recurrent low blood counts and splenomegaly, which may be due to XIAP’s role in regulating caspases or alternative signalling pathways^61^. Interestingly, in the context of the current study, it has been reported that constitutively active mitochondrial C-RAF can also result in mild to moderate splenomegaly^62^. Indeed, expression of a kinase-activating C-RAF mutant, C-RAF(L613V), in a murine model of Noonan syndrome, results in splenomegaly, a phenotype also associated with XIAP-deficient patients^63^. It would be interesting to investigate if the regulation of C-RAF and cell migration by XIAP has any role in the progression of XLP2, either through spleen enlargement or progression of any of the other phenotypes.

Collectively our data reveal that XIAP has a critical role in regulating cell migration through its ability to polyubiquitinate and target C-RAF for proteolytic destruction. These results could provide further clues to the role of XIAP in human disease.

## Supplementary Information

Supplementary Figures.

Supplementary Legends.
