# Supplementary material for: Increased migration and motility in XIAP-null cells mediated by the C-RAF protein kinase
Source: Sci Rep. 2022 May 13;12:7943. doi: 10.1038/s41598-022-11438-8 (PMC9106734; doi:10.1038/s41598-022-11438-8)

Sup Fig 1

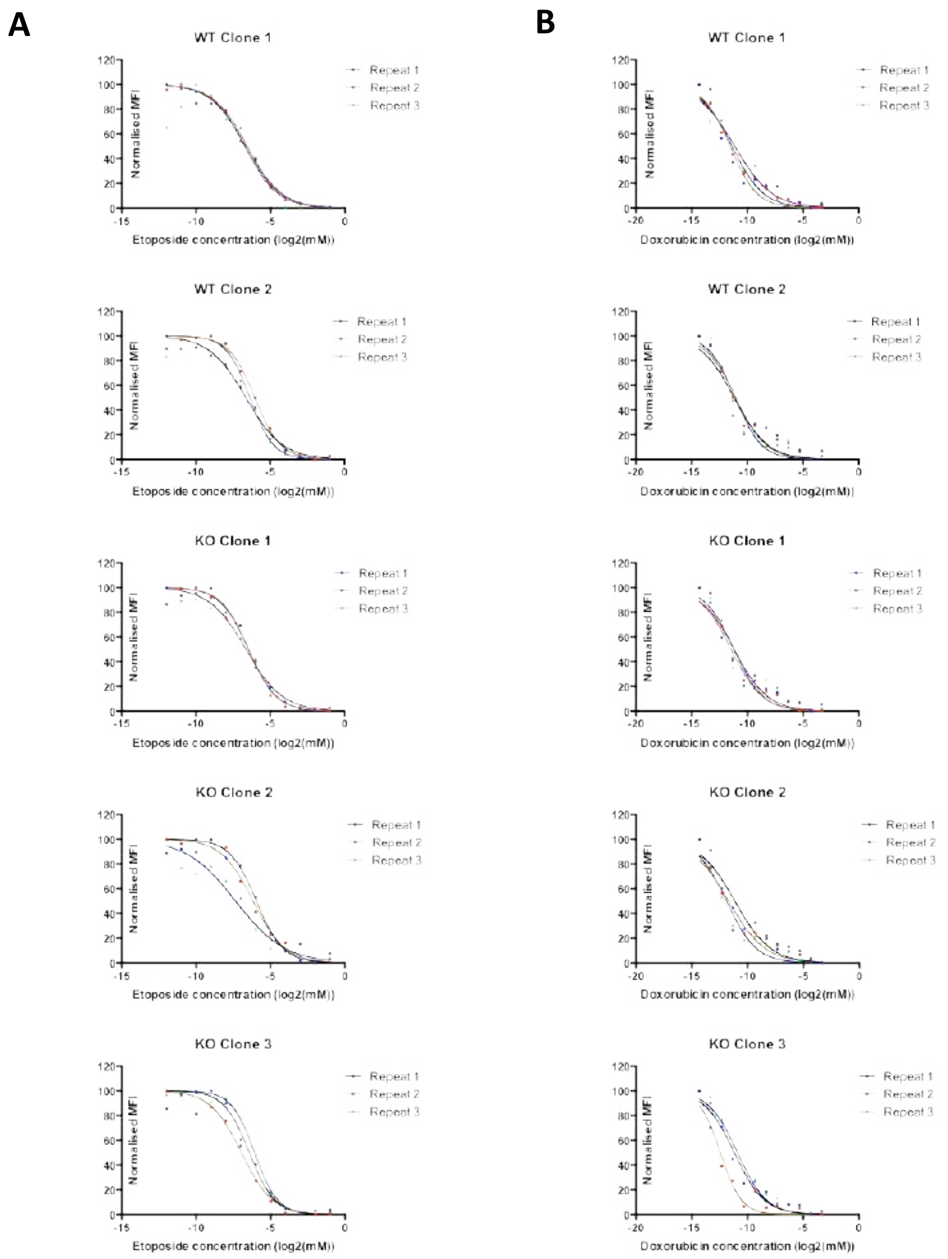
A
B
WT Clone 1
WT Clone 2
KO Clone 1
KO Clone 2
KO Clone 3
Normalised MFI
Repeat 1
Repeat 2
Repeat 3
Etoposide concentration (log2(mM))
Doxorubicin concentration (log2(mM))

Sup Fig 2

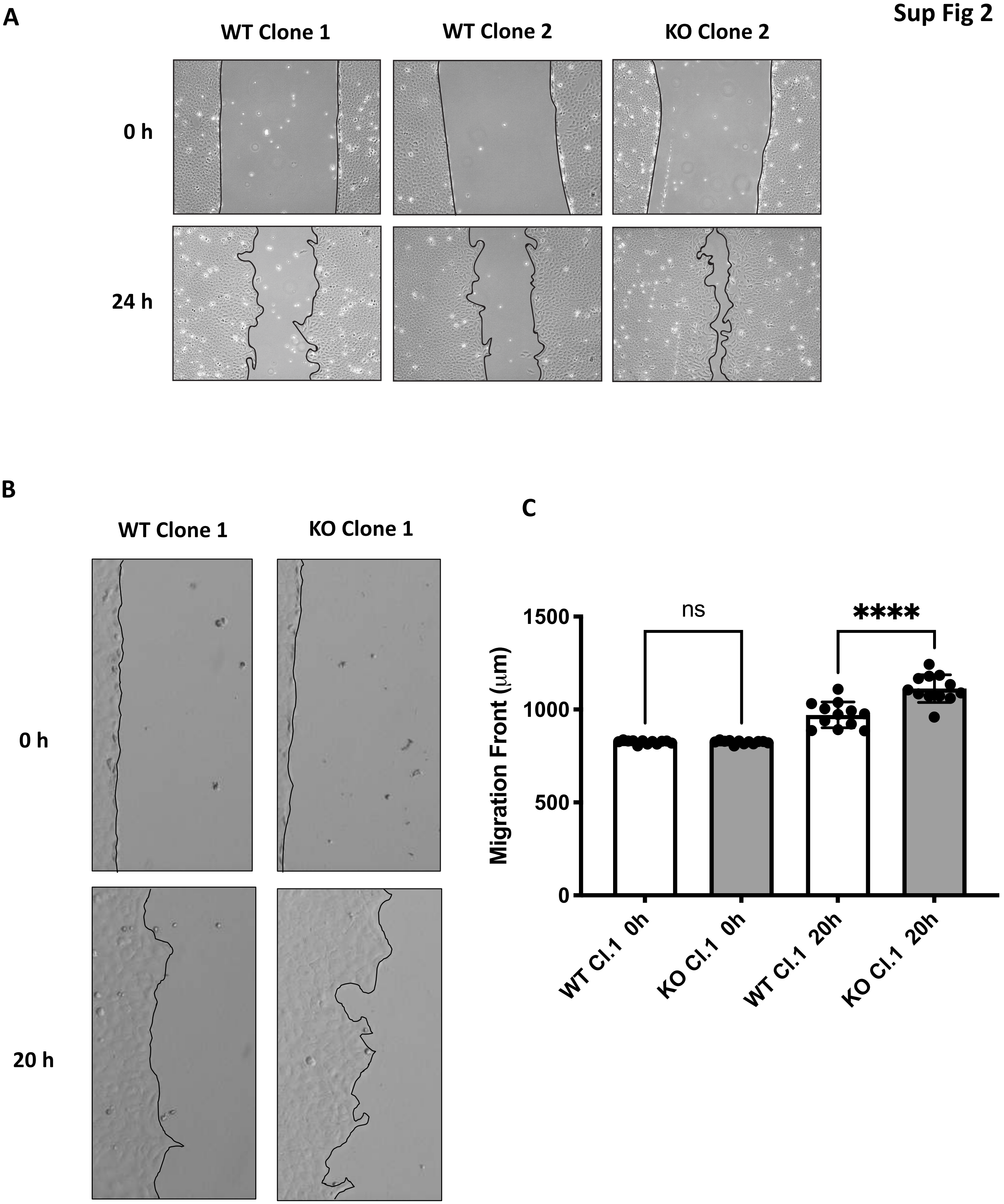
A
WT Clone 1
WT Clone 2
KO Clone 2
0 h
24 h
B
WT Clone 1
KO Clone 1
0 h
20 h
C
ns
****
1500
1000
500
0
Migration Front (μm)
WT Cl.1 0h
KO Cl.1 0h
WT Cl.1 20h
KO Cl.1 20h

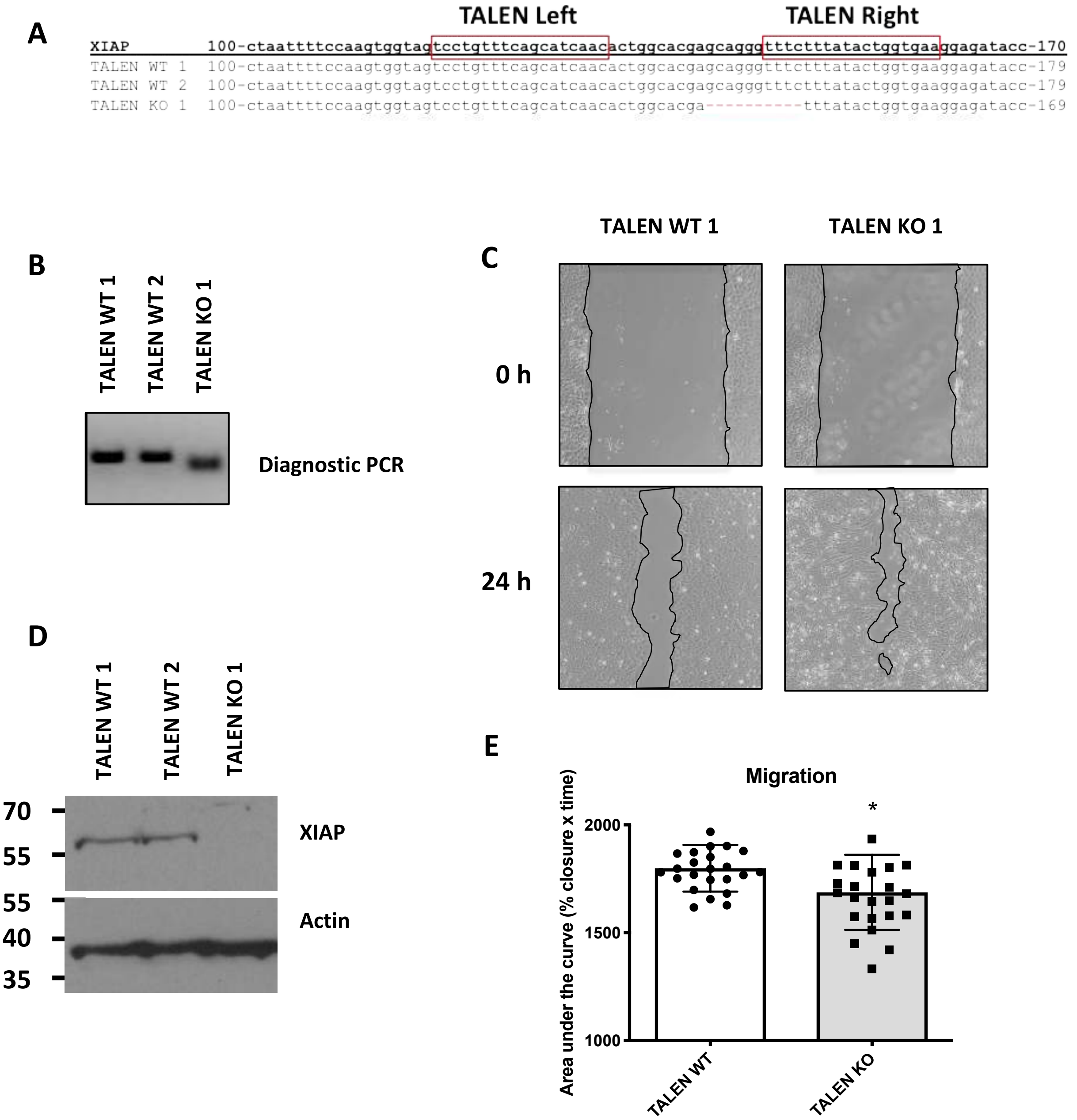
A
TALEN Left
TALEN Right
XIAP 100-ctaattttccaagtggtagtcctgtttcagcatcaacactggcacgagcagggtttctttatactggtgaaggagatacc-170
TALEN WT 1 100-ctaattttccaagtggtagtcctgtttcagcatcaacactggcacgagcagggtttctttatactggtgaaggagatacc-179
TALEN WT 2 100-ctaattttccaagtggtagtcctgtttcagcatcaacactggcacgagcagggtttctttatactggtgaaggagatacc-179
TALEN KO 1 100-ctaattttccaagtggtagtcctgtttcagcatcaacactggcacga----------tttatactggtgaaggagatacc-169
B
TALEN WT 1
TALEN WT 2
TALEN KO 1
Diagnostic PCR
C
TALEN WT 1
TALEN KO 1
0 h
24 h
D
TALEN WT 1
TALEN WT 2
TALEN KO 1
70
55
55
40
35
XIAP
Actin
E
Migration
*
Area under the curve (% closure x time)
2000
1500
1000
TALEN WT
TALEN KO

A

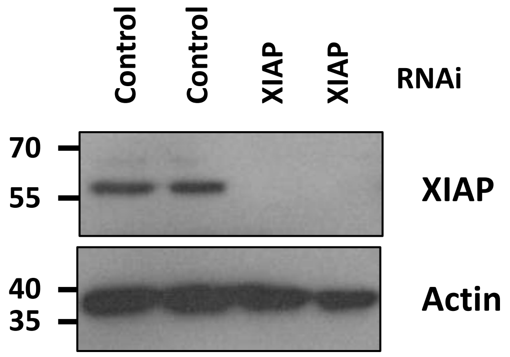
Control
Control
XIAP
XIAP
RNAi
70
55
XIAP
40
35
Actin

B

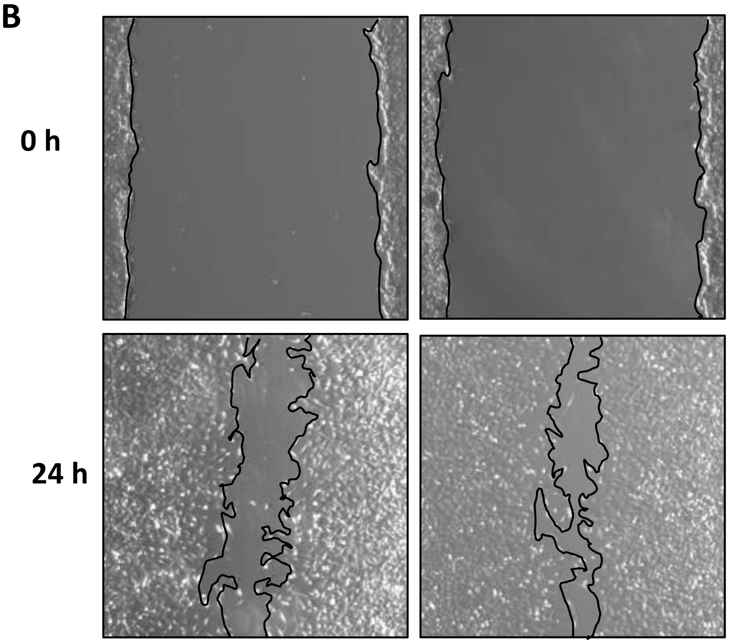
0 h
24 h

C

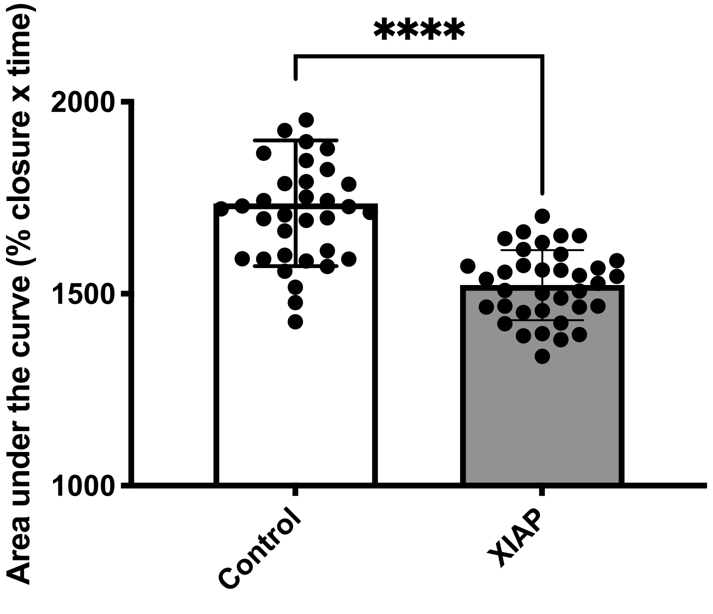
****
Area under the curve (% closure x time)
2000
1500
1000
Control
XIAP

Sup Fig 5

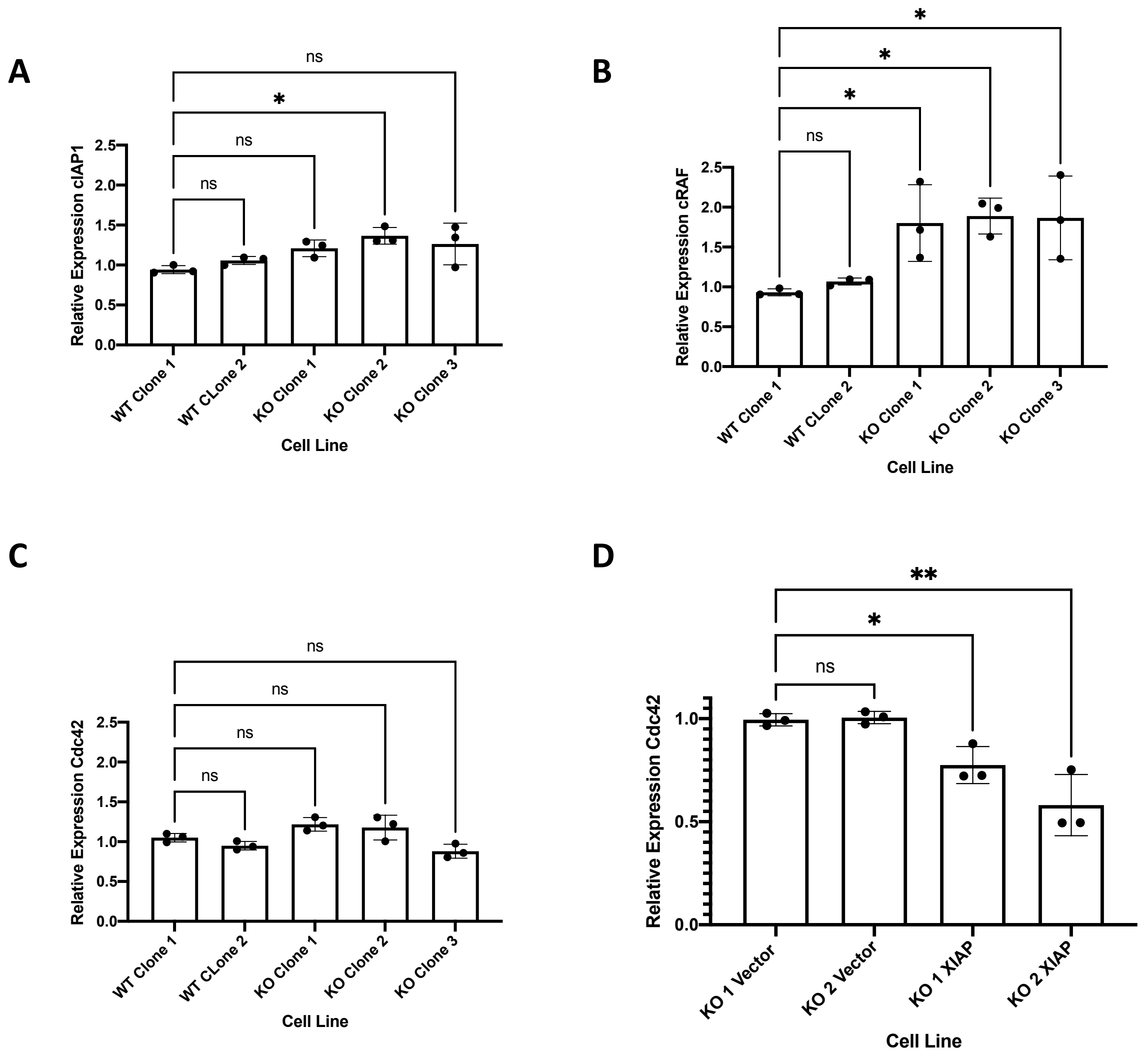

A
Relative Expression cIAP1
WT Clone 1
WT CLone 2
KO Clone 1
KO Clone 2
KO Clone 3
Cell Line
ns
*
B
Relative Expression cRAF
C
Relative Expression Cdc42
D
**
KO 1 Vector
KO 2 Vector
KO 1 XIAP
KO 2 XIAP

ure 1B

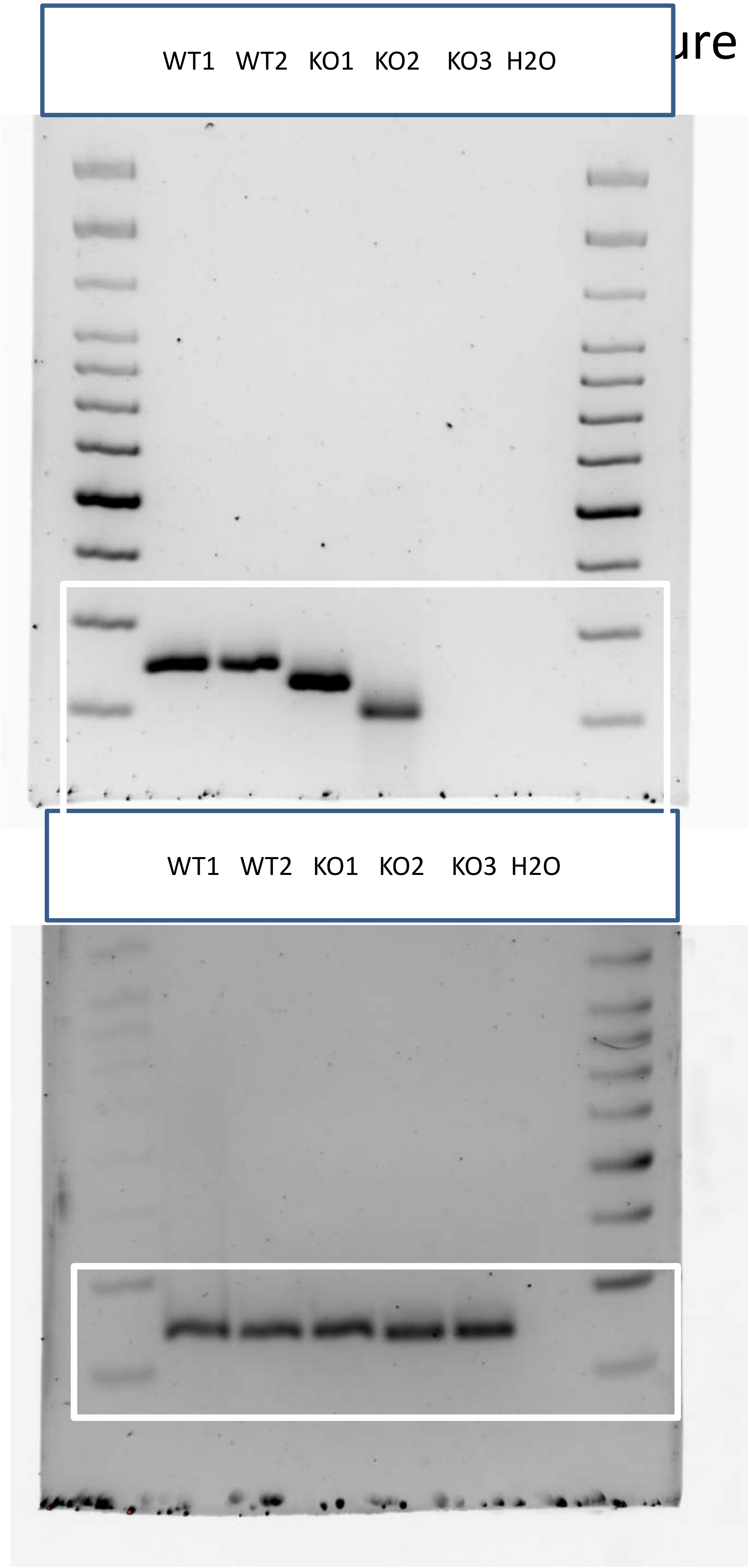

Figure 1D

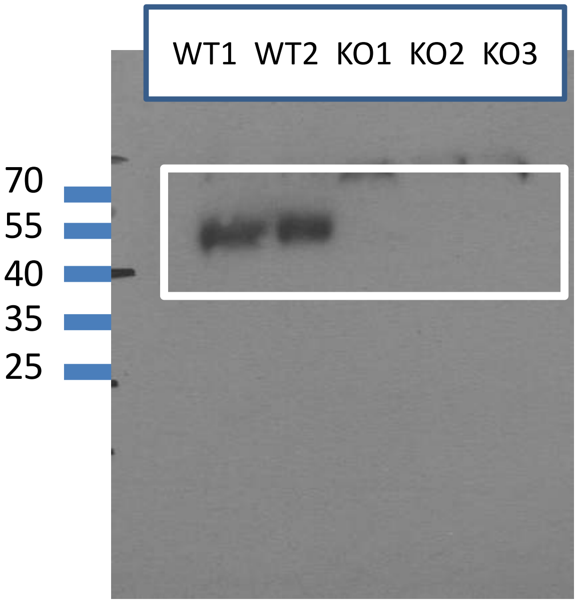
WT1 WT2 KO1 KO2 KO3
70
55
40
35
25

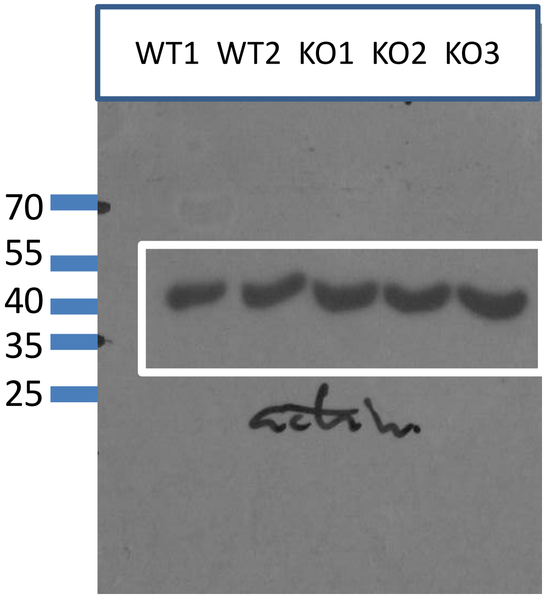
WT1 WT2 KO1 KO2 KO3
70
55
40
35
25
actin

Figure 1E

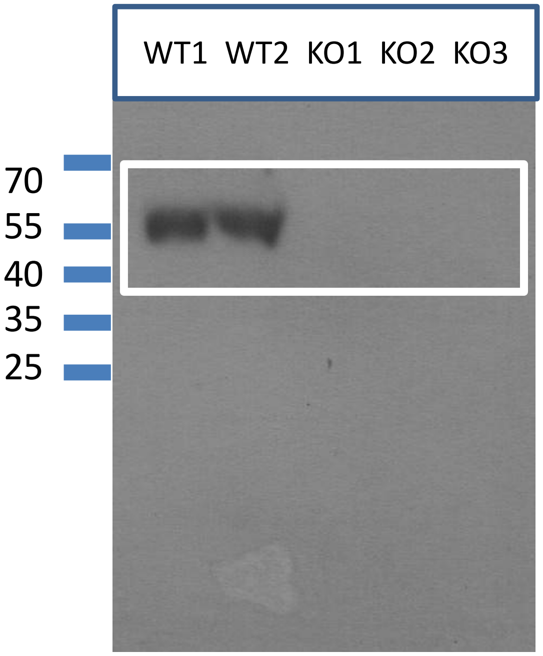
WT1 WT2 KO1 KO2 KO3
70
55
40
35
25

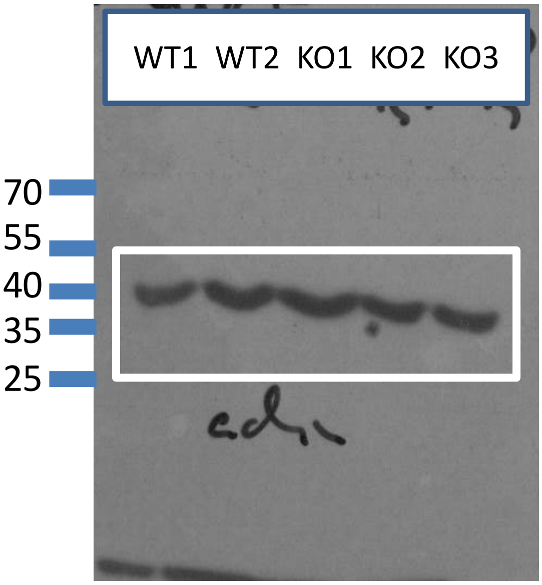
WT1 WT2 KO1 KO2 KO3
70
55
40
35
25

Figure 1E

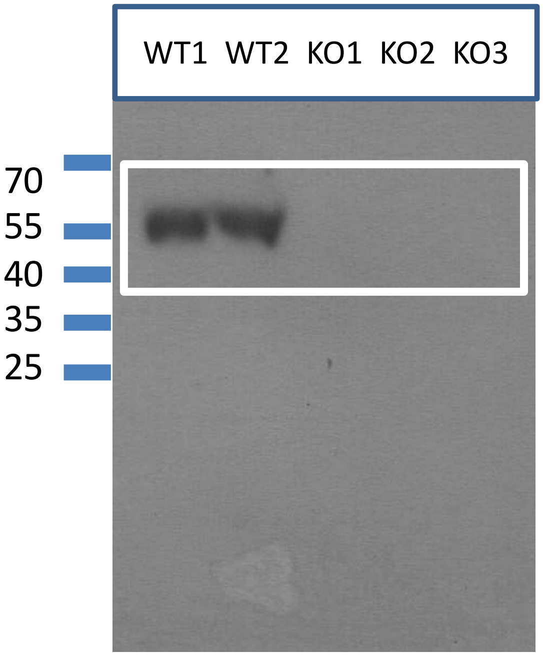
WT1 WT2 KO1 KO2 KO3
70
55
40
35
25

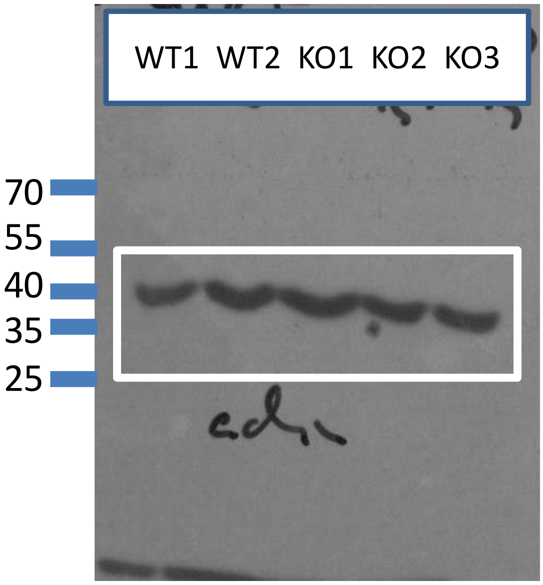
WT1 WT2 KO1 KO2 KO3
70
55
40
35
25

Figure 4A

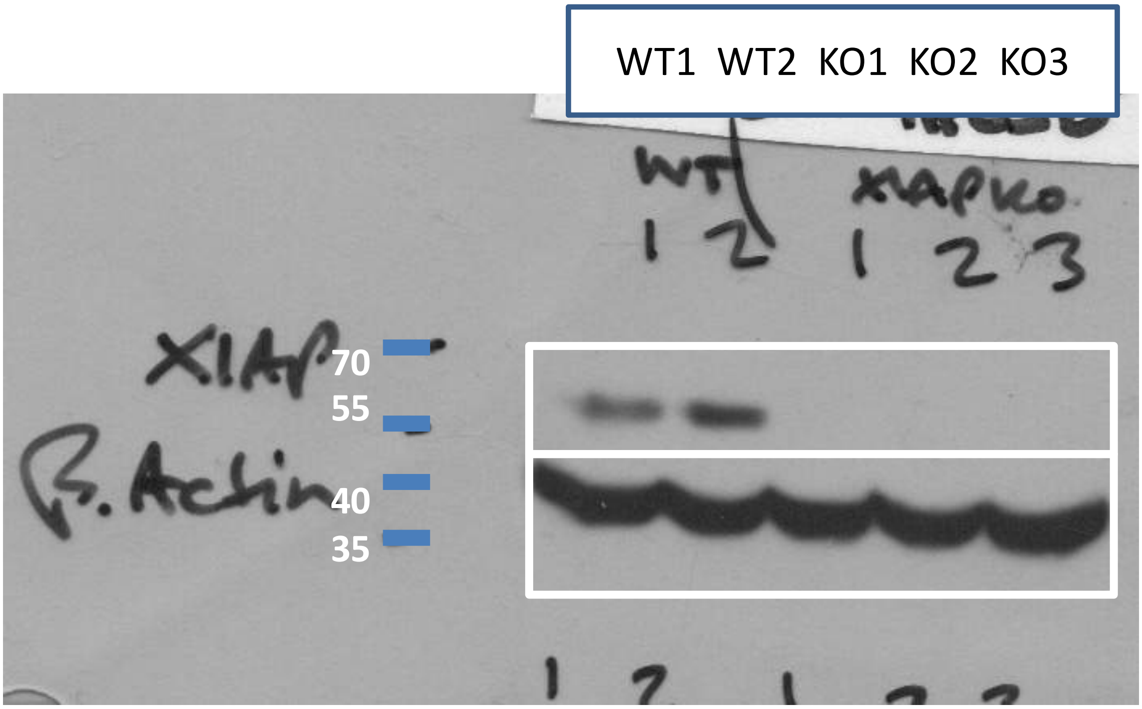
WT1 WT2 KO1 KO2 KO3
70
55
40
35

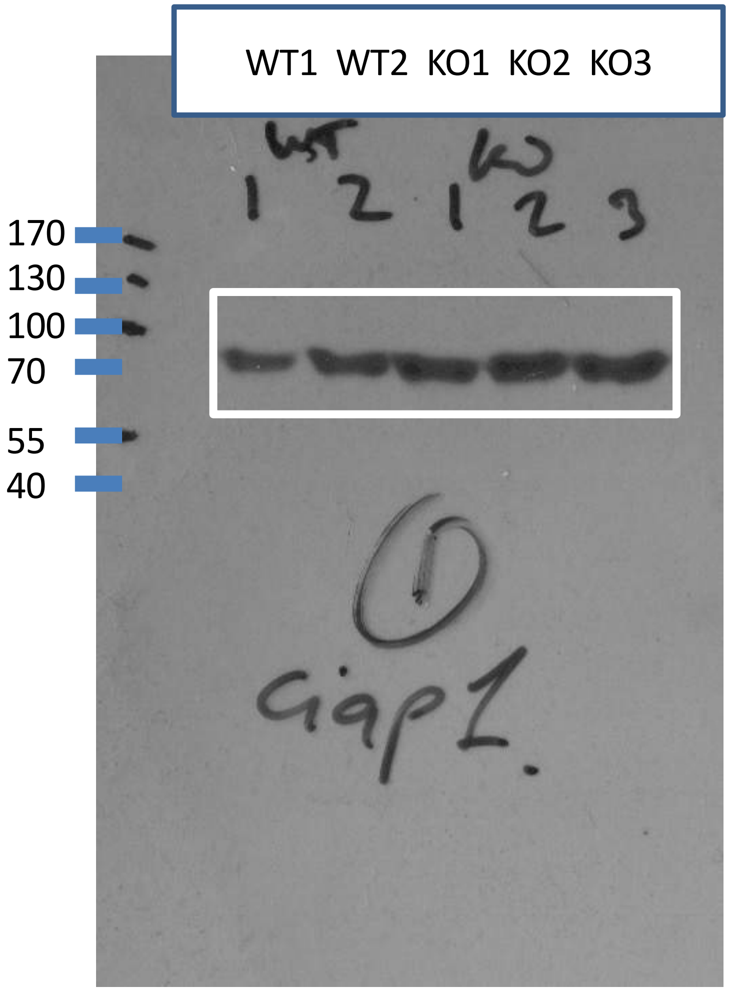
WT1 WT2 KO1 KO2 KO3
170
130
100
70
55
40

Figure 4B

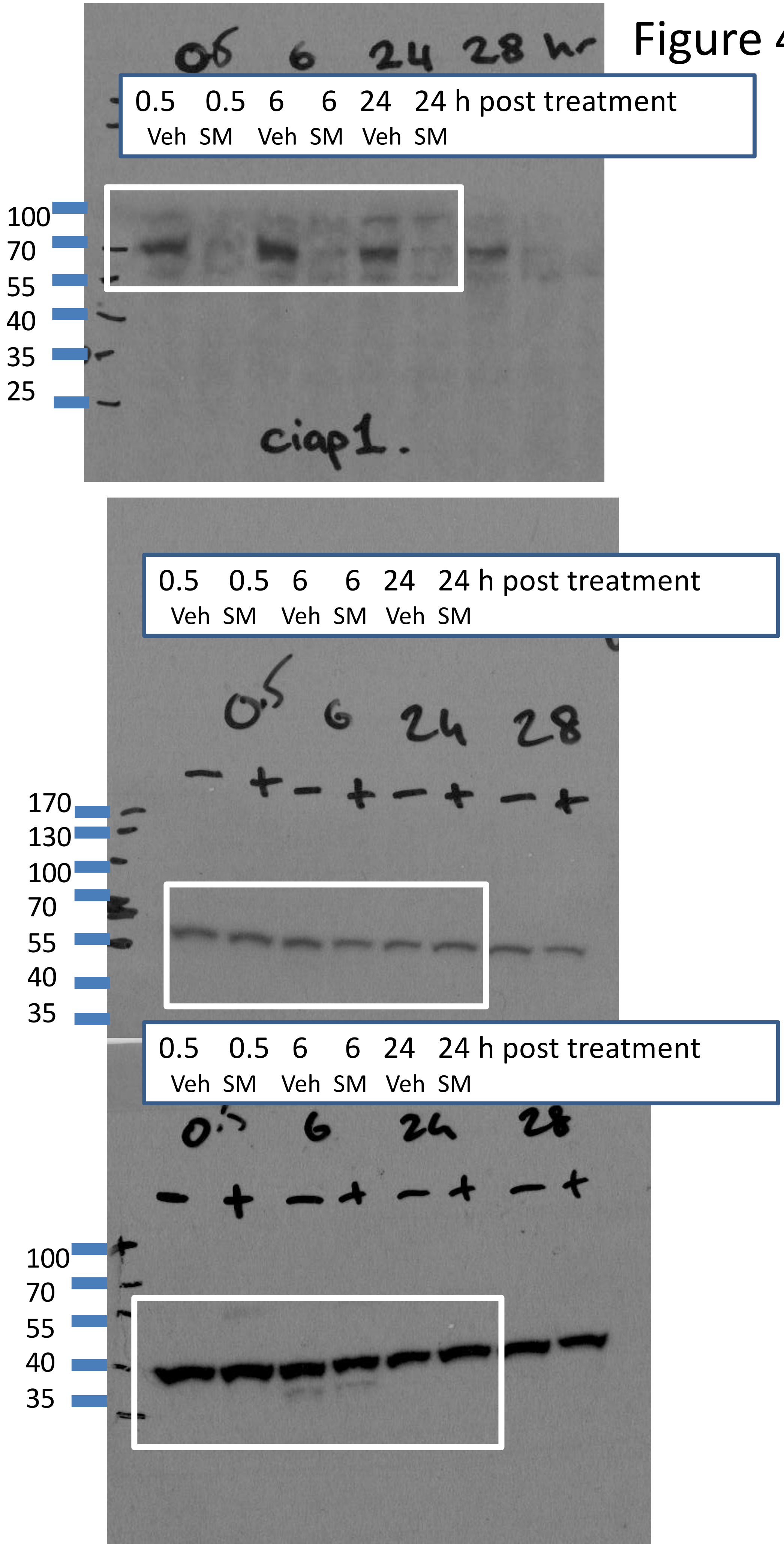

0.5 0.5 6 6 24 24 h post treatment
Veh SM Veh SM Veh SM
100
70
55
40
35
25
ciap1.
0.5 0.5 6 6 24 24 h post treatment
Veh SM Veh SM Veh SM
170
130
100
70
55
40
35
0.5 0.5 6 6 24 24 h post treatment
Veh SM Veh SM Veh SM
100
70
55
40
35

Figure 5B

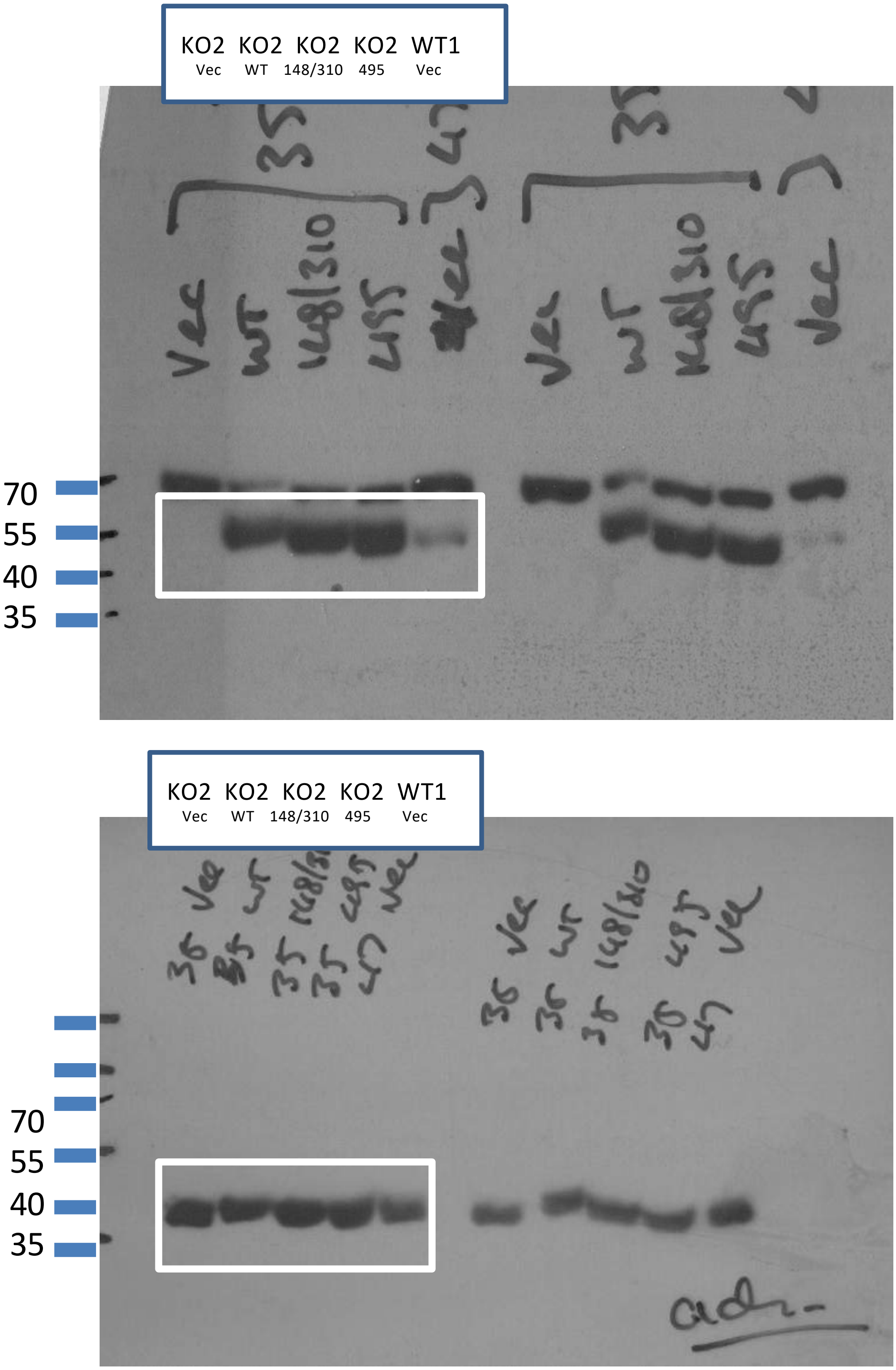
KO2 Vec
KO2 WT
KO2 148/310
KO2 495
WT1 Vec
70
55
40
35
KO2 Vec
KO2 WT
KO2 148/310
KO2 495
WT1 Vec
70
55
40
35

Figure 6A

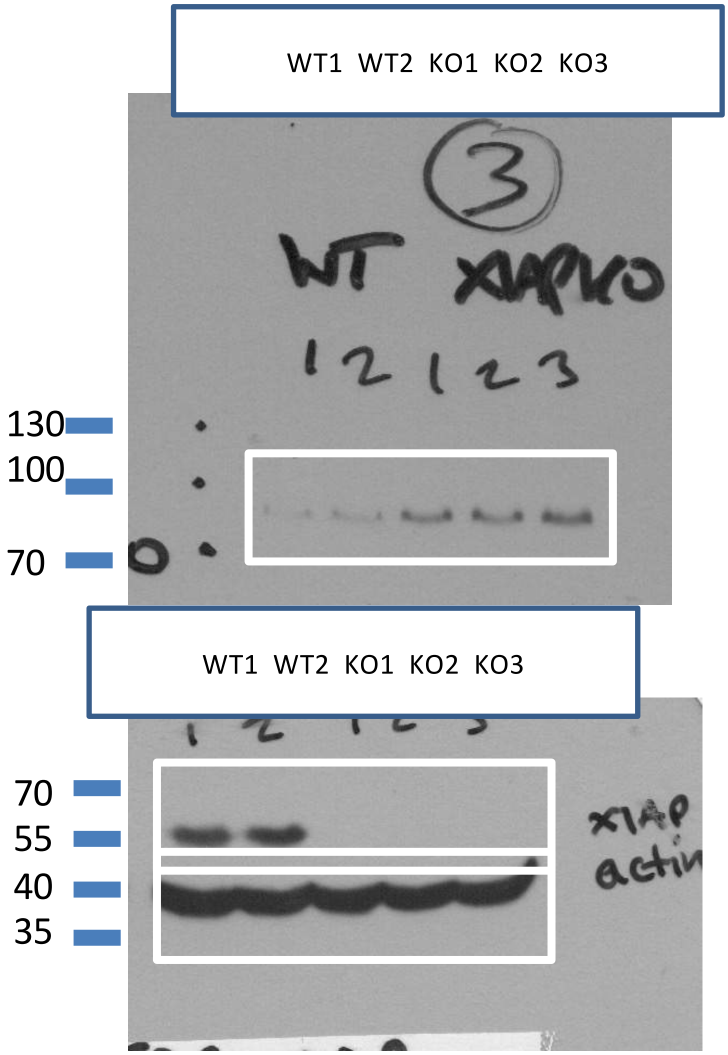

Figure 6B

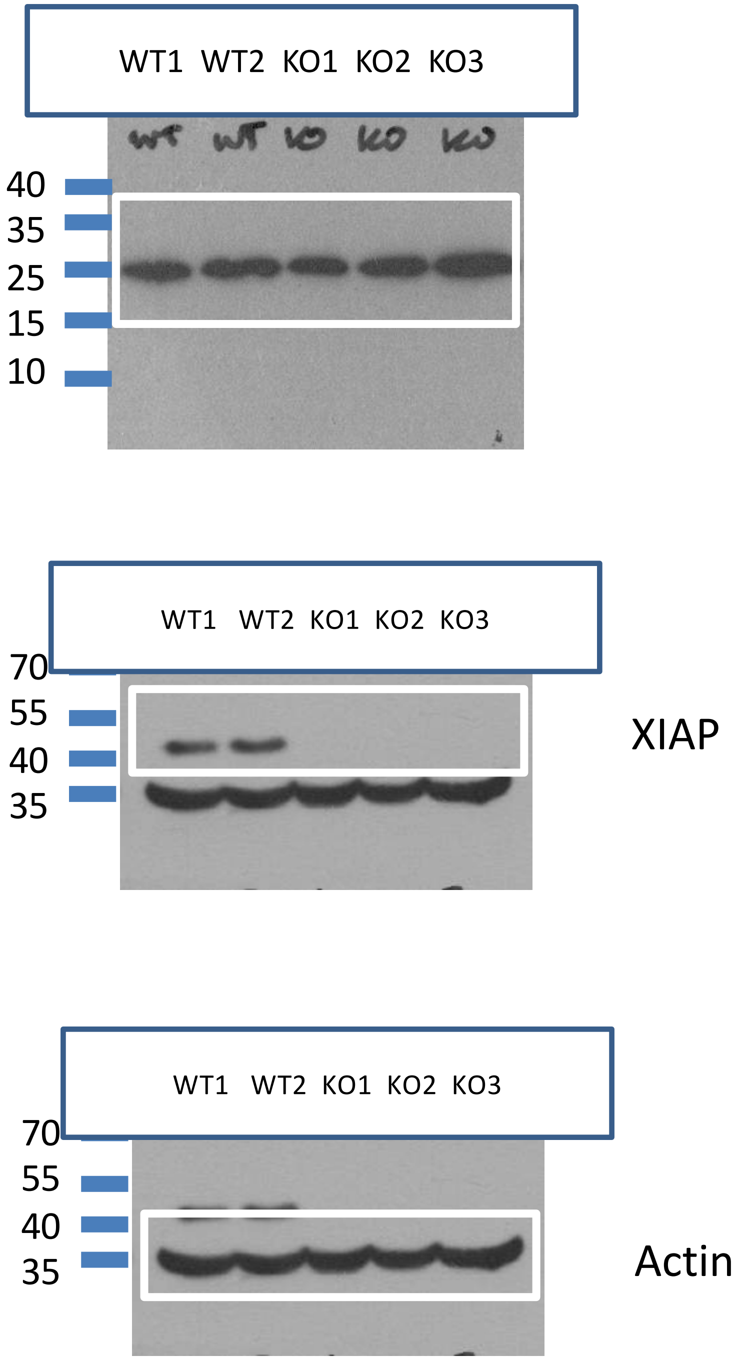

WT1 WT2 KO1 KO2 KO3
40
35
25
15
10
WT1 WT2 KO1 KO2 KO3
70
55
40
35
XIAP
WT1 WT2 KO1 KO2 KO3
70
55
40
35
Actin

Figure 6C

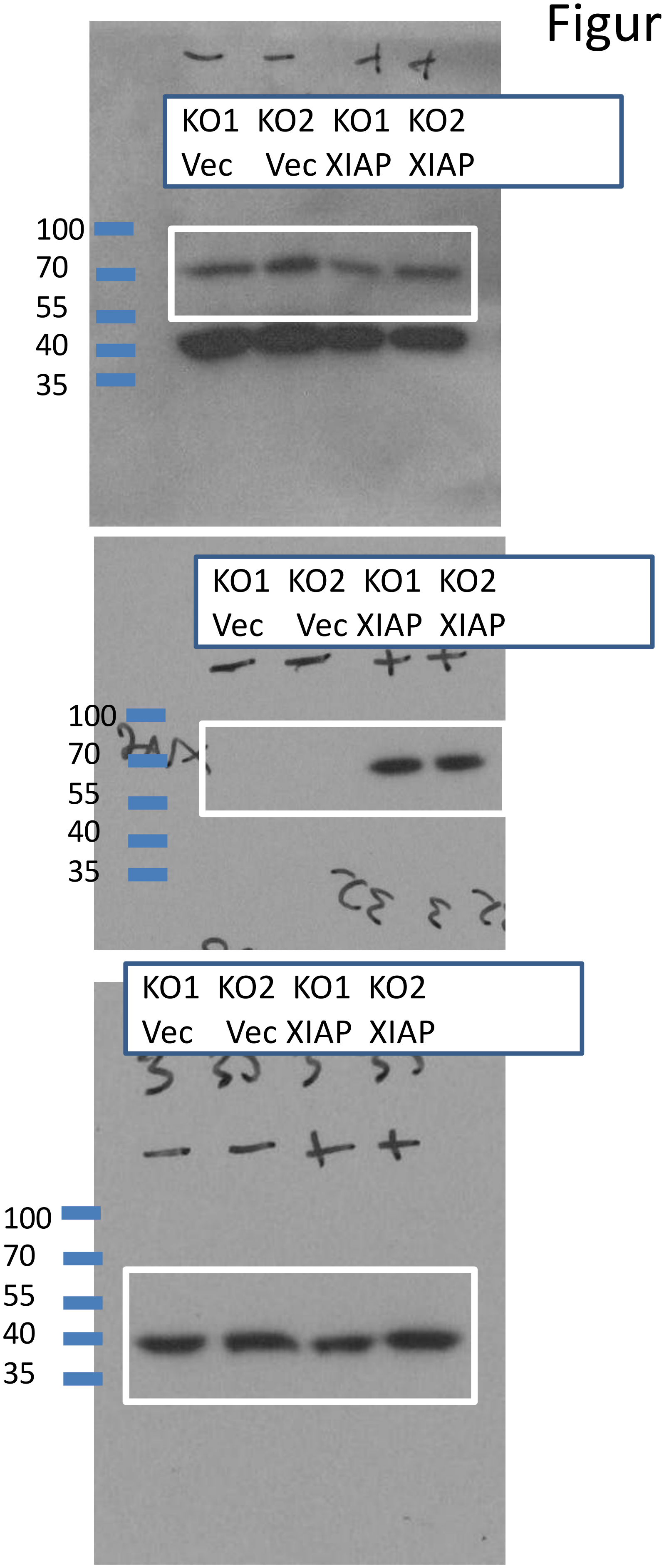
KO1 KO2 KO1 KO2
Vec Vec XIAP XIAP
100
70
55
40
35
KO1 KO2 KO1 KO2
Vec Vec XIAP XIAP
100
70
55
40
35
KO1 KO2 KO1 KO2
Vec Vec XIAP XIAP
100
70
55
40
35

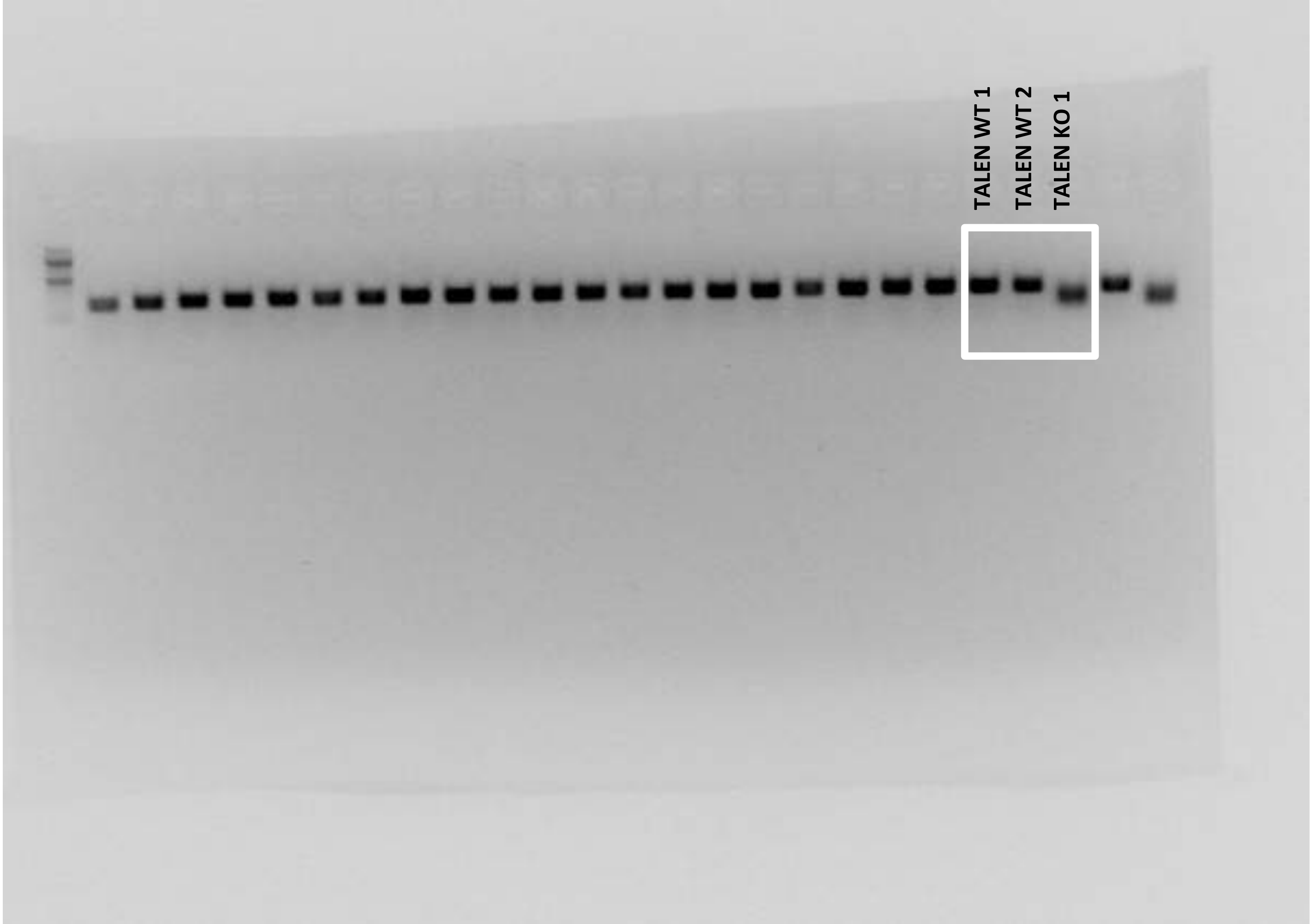

TALEN WT 1
TALEN WT 2
TALEN KO 1

Supplementary Figure 3D

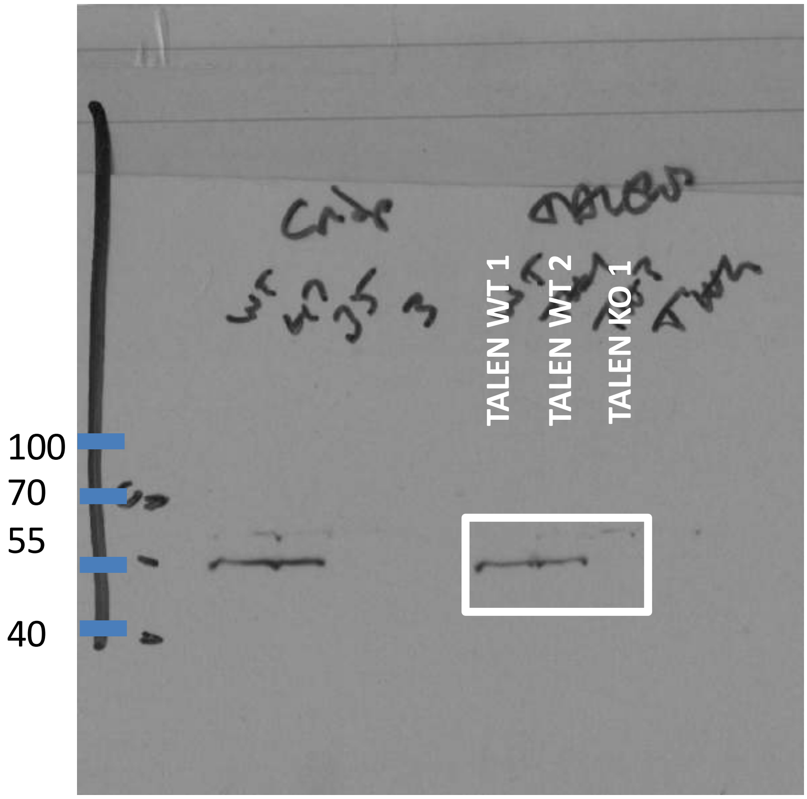

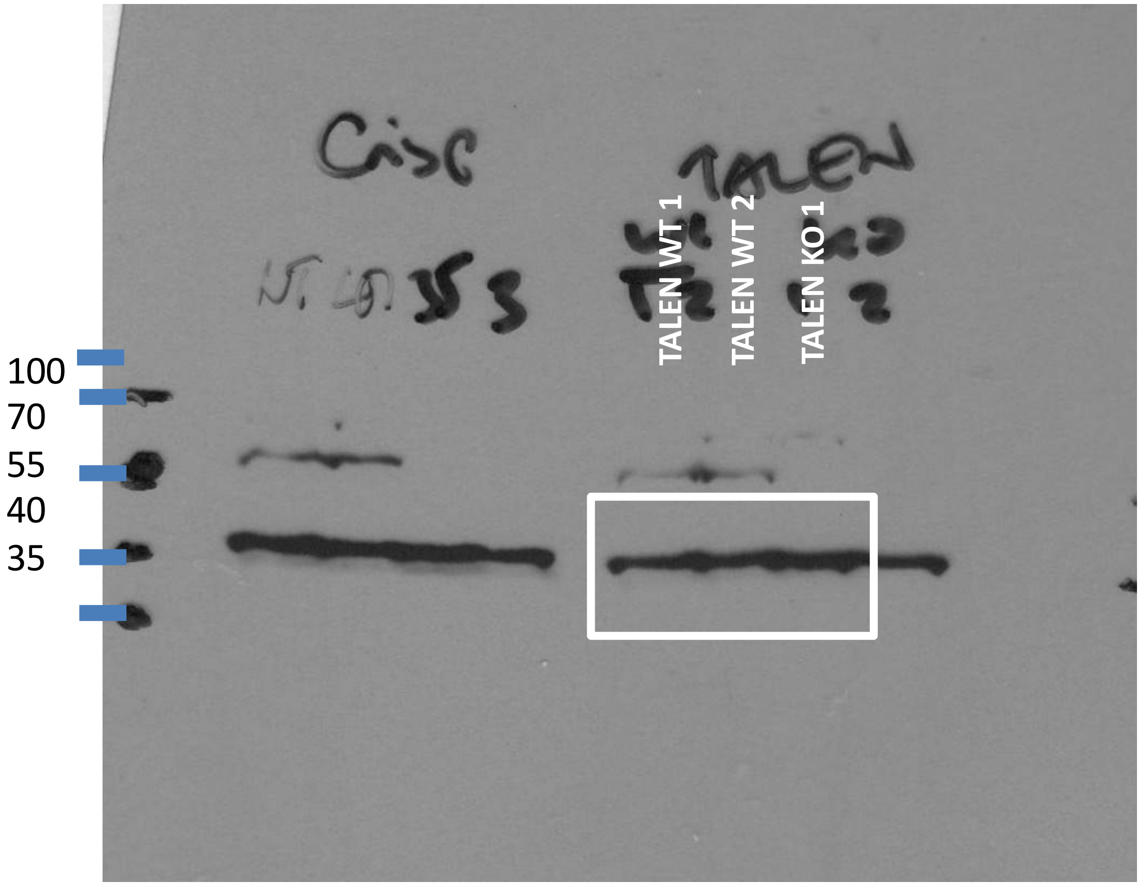

Supplementary Figure 4A

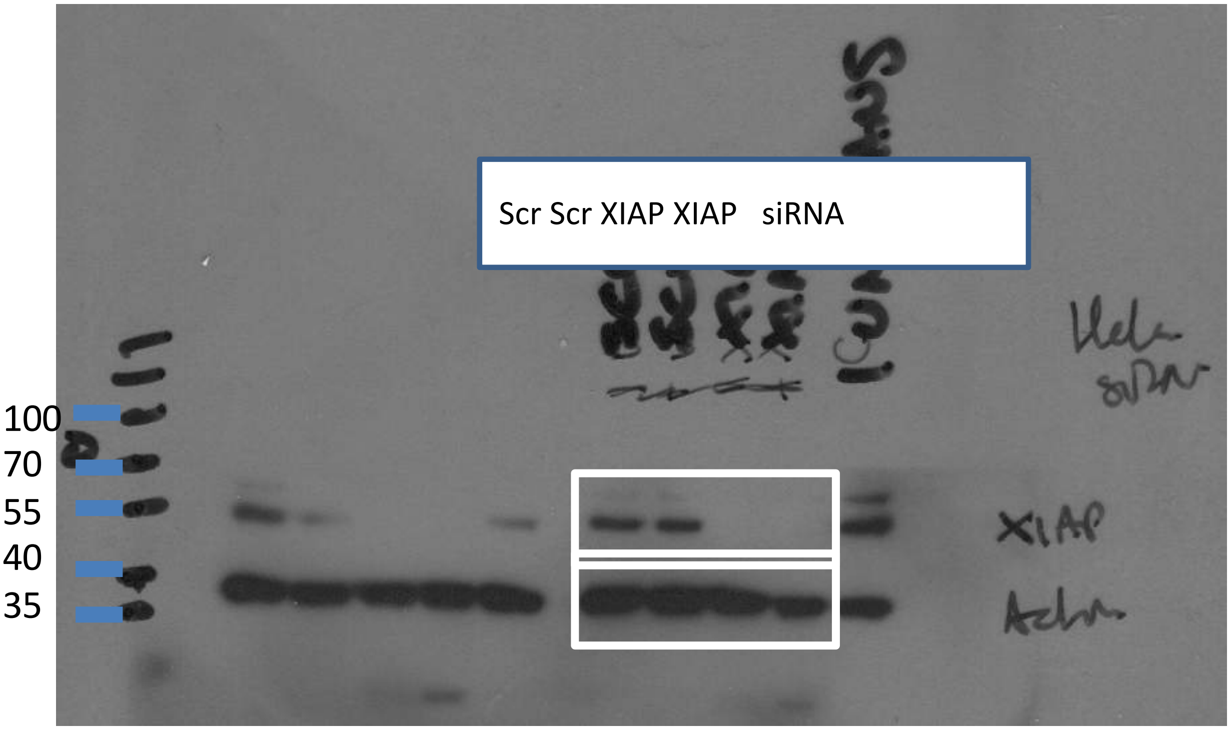

Supplement: Supplementary file 1 — Supplementary Figures. [file 41598_2022_11438_MOESM1_ESM.pdf]
