# Supplementary material for: Increased migration and motility in XIAP-null cells mediated by the C-RAF protein kinase
Source: Sci Rep. 2022 May 13;12:7943. doi: 10.1038/s41598-022-11438-8 (PMC9106734; doi:10.1038/s41598-022-11438-8)
Supplement: Supplementary file 2 — Supplementary Legends. [file 41598_2022_11438_MOESM2_ESM.docx]

**Supplementary Figures**

**Supplementary Figure 1.** **A.** U2OS clonal cell lines were exposed to etoposide for 48 hours and viability was determined by PrestoBlue assay. The fluorescence intensity was measure and each data set were normalised to the reading from untreated cells. The normalised mean fluorescence intensity (MFI) at increasing concentrations of etoposide (log2(mM)) for each clonal cell line is shown. Only data sets with a value of R2>0.85 were used and each curve represents the individual experimental repeats used to calculate the averages used in Figure 2A. **B**. As in (A), but using Doxorubicin as the cytotoxic agent.

**Supplementary Figure 2. XIAP-null cells have increased migratory capacity. A** Representative images of a wound healing experiment for wild type and XIAP null clones at 0 and 24 hours. The migration front is highlighted for clarity. **B** Representative images of the migration front for wild type and XIAP null clones at 0 and 24 hours. C The length of the migration front was measured at 0 and 24hr using Image J software. The migration front length was plotted and analysed by unpaired students t-test.

**Supplementary Figure 3. XIAP-null cells have increased migratory capacity A** DNA sequence of the first exon of the XIAP gene with the TALEN binding sites indicated and allignments of DNA sequencing analysis of TALEN modified clonal U2OS cell lines. **B** PCR analysis of the genomic DNA of TALEN modified U2OS cell line using primers spanning the sites of TALEN-mediated cleavage. **C.** Whole cell lysates were prepared from TALEN-modified U2OS clonal cell lines. Lysates were subjected to immunoblot analysis to assess XIAP expression levels using a specific XIAP antibody (BD). **D** Representative images of a wound healing experiment for wild type and XIAP null clones at 0 and 24 hours. The migration front is highlighted for clarity. **E** The area of the wound was imaged and measured at 0, 8, and 24 hours using ImageJ software. The data was normalised with the percentage wound area equalling 100% at 0 hours. The % wound area was plotted against time and the area under the curve (% wound area x time(hours)) was calculated and analysed by unpaired students t-test.

**Supplementary Figure 4. XIAP-depleted HeLa cells have increased migratory capacity**. **A** HeLa cells transfected with the indicated siRNAs, whole-cell lysates (WCLs) prepared from these cells were subjected to immunoblot analysis to assess expression levels of the indicated proteins. **B** Representative images of a wound healing experiment for control and XIAP-depleted cells at 0 and 24 hours. The migration front is highlighted for clarity. The area of the wound was imaged and measured at 0 and 24 hours using ImageJ software. **C** The data was normalised with the percentage wound area equalling 100% at 0 hours. The % wound area was plotted against time and the area under the curve (% wound area x time(hours)) was calculated and analysed by unpaired students t-test. 24 scratches were measured (8 technical replicates from 3 independent experiments).

**Supplementary Figure 5 XIAP controls levels of C-RAF to influence cell migration.** The relative abundance of proteins **A** cIAP1 **B** C-RAF **C** Cdc42 as compared to b-actin were quantified from 3 independent experiments in XIAP WT and KO cells using ImageJ software. Abundance was normalised to the mean of the 2 WT cell lines. Statistical analysis performed using a one-way ANOVA with Dunnett’s multiple comparisons test and compared to the XIAP WT Clone 1. D. Relative abundance of C-RAF as compared to b-actin was quantified from 3 independent experimetns in XIAP KO cells transfected with empty vector or HA-XIAP. Abundance was normalised to the mean of the XIAP KO cell lines transfected with empty vector. Statistical analysis performed using a one-way ANOVA with Dunnett’s multiple comparisons test and compared to the XIAP KO Clone 1 transfected with empty vector.
